# Electroanatomy of hippocampal activity patterns: theta, gamma waves, sharp wave-ripples, and dentate spikes

**DOI:** 10.3389/fnbeh.2025.1685846

**Published:** 2025-10-23

**Authors:** Nicholas Paleologos, Mihály Vöröslakos, Joaquin Gonzalez, Anna Maslarova, Deren Aykan, Anli A. Liu, György Buzsáki

**Affiliations:** ^1^Neuroscience Institute, Grossman School of Medicine, New York University, New York, NY, United States; ^2^Department of Psychiatry, Grossman School of Medicine, New York University, New York, NY, United States; ^3^Department of Neurology, Grossman School of Medicine, New York University, New York, NY, United States

**Keywords:** hippocampus, electrophysiology, regional interactions, current-source analysis, local field potential, pyramidal cells, interneurons, maps

## Abstract

Monitoring representative fractions of neurons from multiple brain circuits in behaving animals is necessary for understanding how different brain regions interact. Using multishank, high-density recording silicon probes (up to 1,024 sites), we describe the main characteristic LFP patterns in the hippocampus, including sharp wave-ripples (SPW-Rs), dentate spikes (DSs), theta, and gamma oscillations. Our novel observations primarily relate to the distinction between subclasses of SPW-Rs and DSs, as well as their neuronal spiking correlations. In addition to the classical SPW-Rs, initiated in the CA2-3 recurrent collateral system and characterized by a large negative sharp wave (sink) in the mid-CA1 stratum radiatum (SPW-R^Rad^), a small subset of ripples, associated with a sink in CA1 str. lacunosum-moleculare was also observed (SPW-R^LM^). The two types of ripple events differed in frequency, magnitude, and neuronal correlates. CA3 pyramidal neurons were strongly active during SPW-R^Rad^ but not during (SPW-R^LM^). DSs could also be grouped further based on their excitatory inputs from the medial and lateral entorhinal cortex (DS^MEC^ and DS^LEC^), by their impact on their physiological targets, and by the brain states into which they were embedded. Overall, our experiments demonstrate the utility and need for high-density recording of both LFP and spiking activity for the appropriate classification of seemingly similar events. These distinctions relate not only to their neurogenesis but also to their behavioral-cognitive contributions.

## Introduction

Under physiological conditions, multiple brain regions cooperate. Local circuit computations are broadcast to broad areas of the brain. Conversely, local computation is under the control of brain-wide state changes (Fernández-Ruiz et al., 2017; Graves et al., 2012; Modi et al., 2023; Turi et al., 2025). Despite this widely accepted view, experiments on the hippocampus typically report behavior-physiological correlations from a single region only. For more effective interpretation of these relationships, monitoring two or, ideally, all hippocampal regions simultaneously is desirable.

Currently, there are two major techniques available for large-scale recordings of individual neurons in local circuits, each with its characteristic advantages and disadvantages: calcium imaging (Sigl-Glöckner and Seibt, 2019) and wire or nano-machined microelectrodes (‘silicon probes’) (Berényi et al., 2014; Steinmetz et al., 2021). Calcium imaging can record from hundreds to thousands of neurons from a single area and provides dendritic and even spine-level spatial resolution (Manley et al., 2024; Seibt et al., 2017). However, it has a tradeoff between its limited temporal resolution (sufficient to identify spike bursts and, under special circumstances, single spikes) and the size of its field-of-view (often confined to surface recordings). Voltage imaging is a promising recent method; however, the interpretation of the recorded signal requires further refinement (Knöpfel and Song, 2019; Haziza et al., 2025). In contrast, silicon probe recordings with a sufficient number of recording sites offer high temporal resolution of spikes and local field potentials (LFPs), which represent a rich mesoscopic source of neural information (Buzsáki et al., 2012; Watson et al., 2018). This is a clear advantage, as the mesoscopic LFP, with appropriate controls, enables the monitoring of average synaptic current inputs to neurons along with their spikes. These features make silicon probe recordings amenable to input–output analysis. High-density probes can record from multiple cortical and subcortical structures at the spatial resolution of single neurons (Angotzi et al., 2019; Berényi et al., 2014; Jun et al., 2017; Steinmetz et al., 2021).

Every neural recording method is a compromise between breadth and depth. Single-shank probes with multiple recording sites at neuron-size intervals (<40 μm) are designed to resolve individual neurons within local populations and offer current source density analysis of LFP. However, there is a lack of spatial coverage. To record from other areas of the same or different structures, insertion of multiple probes is needed. Probes with multiple shanks can record from a large volume of neuronal tissue (Berényi et al., 2014; Seymour et al., 2017; Williams et al., 2025), providing breadth but at the expense of resolving single neurons with high fidelity. A solution to these competing requirements is to fabricate multishank probes with high-density sites. Four-shank Neuropixels-2.0 probes are in this direction; yet, despite the large number of potential recording sites, their ability to simultaneously monitor neuronal activity is currently confined to <400 sites (Jun et al., 2017; Steinmetz et al., 2021).

To provide recordings from all regions of the dorsal hippocampus with high spatial resolution, we sampled the extracellular space with SiNAPS multishank probes. These probes enable simultaneous recordings from 1,024 sites continuously, covering an area of 2 mm × 2 mm, distributed across 8 shanks, with an inter-shank distance of 300 μm (Angotzi et al., 2019, 2025). Our goal is to provide high spatial resolution, two-dimensional images of the major hippocampal network patterns, including theta and gamma oscillations, sharp wave-ripples (SPW-Rs), and dentate spikes (DS) to the hippocampus community. These maps will enable investigators to embed their physiological recordings using low-density probes and tetrodes, providing a reliable anatomical reference for online physiological recordings without the need for histological verification of the probes. In several additional experiments, we compare the data obtained with the SiNAPS multishank probes with Neuropixels 2.0 and high-density single-shank silicon probe recordings. Our current work is an update of several previous experiments to relate electrophysiological fingerprints to anatomical, hippocampal layer boundaries (Table 1) (Angotzi et al., 2025; Berényi et al., 2014; Bragin et al., 1995a; Bragin et al., 1995b; Csicsvari et al., 2003; Einevoll et al., 2013; Kajikawa and Schroeder, 2015; Mackey et al., 2024; Schroeder et al., 1998; Senzai and Buzsáki, 2017; Sullivan et al., 2011, 2014).

**Table 1:** Summary of recordings illustrating hippocampal electrophysiological phenomena.

Figure #	Phenomenon illustrated	Electrode type	Behavioral state	Animals/sessions used	Relevant studies
1	Hippocampal layer identification based on electrophysiology	SiNAPS (head-fixed)	Awake, NREM sleep	(b) NN_syn_M10: (d) NN_syn_M02: 6/7/23 session	Andersen et al., 1973; Witter et al., 1989; Schomburg et al., 2014; Senzai and Buzsáki, 2017; Fernández-Ruiz et al., 2021; Santiago et al., 2024; Angotzi et al., 2025;
2	CSD maps of hippocampal activity patterns (theta, SPW-Rs, and DSs)	SiNAPS (head-fixed)	Awake (theta), NREM sleep (SPW-Rs, DS)	NN_syn_M02: 6/7/23 session	Buzsáki et al., 1986; Brankack et al., 1993; Buzsáki, 2002; Sullivan et al., 2011, 2014; Stark et al., 2014; Zutshi et al., 2022
3	Coherence maps (theta, gamma, and ripple band)	SiNAPS (head-fixed)	Awake, NREM sleep	NN_syn_M02: 6/7/23 session	Csicsvari et al., 2003; Montgomery et al., 2008, 2009; Berényi et al., 2014;
4	Theta and gamma power maps	SiNAPS (head-fixed)	Awake	NN_syn_M02: 6/7/23 session	Winson, 1974; Buzsáki et al., 1986; Lubenov and Siapas, 2009; Zutshi et al., 2022
5	Phase-amplitude coupling	SiNAPS (head-fixed)	Awake	NN_syn_M02: 6/1/23 session	Colgin et al., 2009; Belluscio et al., 2012; Schomburg et al., 2014; Sullivan et al., 2014; Fernández-Ruiz et al., 2017; Lopes-dos-Santos et al., 2025
6	CSD-based classification of SPW-Rs	SiNAPS (head-fixed)	NREM sleep	NN_syn_M02: 6/7/23 session	Sebastian et al., 2023; Castelli et al., 2025
7	CSD-based classification of dentate spikes	NP2.0 (freely-moving)	Awake (linear maze), NREM sleep (homecage)	HPC_TR_M01: 6/12/25 session	McNaughton and Barnes, 1977; Bragin et al., 1995a; Bragin et al., 1995b; Farrell et al., 2024; McHugh et al., 2024;
8	CSD-based DS^LEC^ clusters	NP2.0 (freely-moving)	Awake (linear maze), NREM sleep (homecage)	HPC_TR_M01: 6/12/25 session	Brankack and Buzsáki, 1986; Farrell et al., 2024; McHugh et al., 2024
9	CSD-based DS^MEC^ clusters	NP2.0 (freely-moving)	Awake (linear maze), NREM sleep (homecage)	HPC_TR_M01: 6/12/25 session	Brankack and Buzsáki, 1986; Dvorak et al., 2021; Farrell et al., 2024; McHugh et al., 2024

The recording and electrode type, behavioral state, and representative session used for the primary electrophysiological features analyzed in each main figure are summarized here. The most relevant studies for the features illustrated in each figure are included in the last column.

## Materials and methods

### Animal experiments

All animal procedures were performed by institutional guidelines and approved by the Institutional Animal Care and Use Committee at New York University Medical Center (license # IA15-01466). The datasets used in this study were previously published in Angotzi et al. (2025) and Zutshi et al. (2022), where detailed descriptions of surgical procedures, animal care, and data acquisition protocols are provided. Across the different studies, we used *n* = 3 (SiNAPS), *n* = 4 (1-shank probe), and *n* = 1 (dual 4-shank Neuropixels 2.0) mice. In brief, adult mice (C57/Bl6 mice, 26–31 g) were implanted with a 3D-printed headpost (Osborne and Dudman, 2014) and head-fixed, awake recordings were performed using 1,024-channel, 8-shank SiNAPS probes (Angotzi et al., 2019; Angotzi et al., 2025) targeting the dorsal hippocampus. A different set of mice were implanted chronically with a single-shank silicon probe and recordings were performed in freely moving conditions (Zutshi et al., 2022). All animals were housed in a controlled environment with a 12-h light/dark cycle and ad libitum access to food and water. In addition, two 4-shank Neuropixels 2.0 probes attached to a microdrive (Vöröslakos et al., 2021) were chronically implanted in a mouse to target the dorsal hippocampus (coordinates relative to bregma: AP -2 mm, ML: −0.75 mm (probe 1) and −1.5 mm (probe 2), DV: −4 mm, 2-degree angle in the AP direction). Prior to implantation, the mouse was habituated to a linear maze (110 cm length × 5 cm width) for three days while following a water-restriction schedule. After five days of recovery from surgery, water restriction was reinstated 24 h prior to the start of behavioral experiments and freely moving recordings. Each session consisted of 1.5 h of homecage sleep, followed by ~1 h on the linear maze, and post-behavioral homecage sleep for 1.5 h. Raw data were acquired at either 20 kHz with the Neuronexus SiNAPS Interface Box or 30 kHz with the SpikeGLX software and OneBox acquisition system, respectively.

### Sleep state scoring and LFP analysis

The LFP signal used for data analysis was obtained by downsampling the raw data to 1,250 Hz. Brain state scoring was performed as described previously (Watson et al., 2016). In short, spectrograms were constructed with a 1-s sliding 10-s window fast Fourier transform of the 1,250 Hz LFP at log-spaced frequencies between 1 Hz and 100 Hz. Three types of signals were used to score states: broadband LFP, narrowband high-frequency LFP, and electromyogram (EMG) calculated from the LFP. For the broadband LFP signal, principal component analysis was applied to the Z-transformed (1–100 Hz) spectrogram. The first principal component in all cases was based on power in the low (32 Hz) frequencies. Dominance was taken to be the ratio of the power at 5–10 Hz and 2–16 Hz from the spectrogram. All states were inspected and curated manually, and corrections were made when discrepancies between automated scoring and user assessment occurred.

### Ripple detection

Ripples were detected using previously described methods (Tingley and Buzsáki, 2020). Briefly, the broadband LFP (1,250 Hz) was filtered in the 120–200 Hz band (Butterworth; order = 3) and transformed to a normalized square signal (NSS). Ripples peaks were identified by the signal exceeding 5 standard deviations above the mean NSS. The start and end timestamps of the ripple were defined by a threshold of 2 standard deviations above the mean NSS. Ripple duration limits were between 15 ms and 100 ms. The EMG signal, estimated from the broadband LFP, was used to exclude artifactual events resulting from high-coherence, EMG-related noise. Ripples were detected across the entire session, including periods on the maze and homecage recordings for chronically implanted mice.

### Dentate spike detection

Dentate spikes were detected using the methods described in Santiago et al. (2024).^1^ Briefly, we detected the peaks of the filtered LFP (1–200 Hz, Butterworth; order = 4) in the channels covering the dentate gyrus (DG). A threshold of 6 times the median absolute amplitude of the filtered LFP was employed, and we only considered events that were at least 50 ms apart.

### Detection of high-theta power epochs

High-theta epochs were identified to isolate periods of elevated theta oscillatory activity for state-specific analyses. Local field potential (LFP) signals were filtered in the theta band (5–12 Hz) using a zero-phase bandpass filter. Theta power was computed using a multi-taper spectrogram (window: 4 s, step: 2 s) from a reference channel located in the stratum oriens, where theta amplitude is typically high and stable. The instantaneous theta power time series was smoothed and thresholded to define high-theta periods. Specifically, time bins in which theta power exceeded the 90th percentile of the full-session theta power distribution was labeled as high-theta. This binary classification was used to restrict subsequent analyses to periods of reliable and sustained theta oscillations.

### Current source density analysis

To visualize the laminar pattern of transmembrane currents surrounding hippocampal activity patterns, we computed the event-triggered average current-source density (CSD). For each event, the broadband LFP was extracted from all channels of a vertical column on a given shank within a time window of ±100 ms (for SPW-Rs and dentate spikes) and ±200 ms around the event peak (for theta oscillations). To correct for slow baseline fluctuations or drifts specific to each event, a linear trend was removed from each LFP snippet individually (*detrend* function in MATLAB). The detrended LFP snippets for each event were then averaged to produce a single event-triggered average LFP matrix for each shank.

The CSD was then estimated from this event-triggered average LFP. As per convention, the polarity of the average LFP was first inverted. The inverted signal then underwent a two-step smoothing process: first, a temporal smoothing for each channel using a Savitzky–Golay filter (*sgolay* function in MATLAB) with a frame length of 11 samples, followed by a spatial smoothing across channels using locally weighted scatterplot smoothing (*lowess* function in MATLAB) with a span of 11 channels. Finally, the CSD was computed as the second spatial derivative of the smoothed LFP (*Φ*), approximated by the second-order difference across adjacent channels using MATLAB’s *diff* function:


$$\mathrm{CSD}(z)\approx -\partial^{2}\Phi /\partial z^{2}\approx -\partial [\Phi (z+\mathrm{\Delta z})-2\Phi (z)+\Phi (z-\mathrm{\Delta z})].$$

σ reflects the medium conductivity, which is assumed to be unitary for simplicity. Here, z represents the vertical depth along the probe, and Δz is the inter-channel spacing.

### Hippocampal layer identification

Hippocampal layers were identified with physiological landmarks derived from local field potential (LFP) CSD profiles. Briefly, LFP signals were filtered into the ripple (120–200 Hz), theta (5–12 Hz), and dentate spike (1–200 Hz) bands. Ripples, theta waves, and dentate spikes were detected as described above. Event peaks for each activity pattern were used as reference timestamps for computing linear CSD profiles. For each shank, the location of the stratum pyramidale (pyramidal layer) was determined based on the channel with the strongest current source in the average SPW-R CSD profile, corresponding to the site with maximal ripple power. The large current source and ripple power reflect dense firing and high-frequency oscillations during SPW-Rs. The middle of the stratum oriens and stratum radiatum were identified by the strongest current sinks immediately above and below the pyramidal layer, respectively. These sinks corresponded to the basal and apical dendrites whereby large excitatory inputs to pyramidal cells are received. Theta CSD profiles referenced to theta peaks detected from the CA1 stratum oriens or stratum pyramidale channels were used to locate the stratum lacunosum-moleculare (LM) on each shank. The site with the largest current sink below the CA1 pyramidal layer identified the middle of the LM layer. The characteristic sink-source phase reversal present in the average dentate spike-triggered CSD profile was used to identify the channels in the mid-molecular layer and granule cell layer of the dentate gyrus. Specifically, the mid-molecular layer was identified by the strongest current sink below the LM layer, whereas the granule cell layer corresponded to the strongest current source immediately below the molecular sink. Identified layer markers were individually assigned to each shank and used to anchor subsequent laminar analyses of spiking activity, CSD, and theta–gamma coupling.

### Classification of DS^LEC^ and DS^MEC^

To classify DS^LEC^ and DS^MEC^, the current source density at the peak of each dentate spike was concatenated into an NxM matrix (containing N electrodes and M dentate spikes). Next, we applied PCA to reduce the dimensionality, yielding a 1xM vector, and then used Gaussian Mixture Models to assign each event in the vector to one of two clusters. This resulted in two distinct event classes with a differential sink profile in the molecular layer. The cluster with the main sink corresponding to the highest channel in DG was classified as DS^LEC^, while the other cluster was classified as DS^MEC^.

### Phase amplitude coupling

To quantify phase–amplitude coupling (PAC) across the hippocampal laminae, we computed the relationship between theta phase (6–10 Hz) and gamma amplitude (30–80 Hz) for each recording channel. Raw local field potentials (LFPs) were filtered in the theta and gamma bands using zero-phase bandpass filters. The theta phase was extracted from the Hilbert transform of the theta-filtered signal, and the gamma envelope (amplitude) was computed from the Hilbert transform of the gamma-filtered signal. Theta phase values were binned into 18 equally spaced intervals between –*π* and π. Gamma amplitudes were averaged within each bin to create a phase–amplitude histogram per channel. Channels were grouped by shanks based on known probe geometry. For each shank, we plotted gamma amplitude as a function of theta phase and electrode depth. Color scales were matched across shanks to enable direct comparison. We computed the Modulation Index (MI) for each channel using the method of (Tort et al., 2010), which measures the Kullback–Leibler divergence between the observed phase–amplitude distribution and a uniform distribution. This provided a scalar estimate of coupling strength per channel. MI values were plotted across depth and compared across shanks.

### Independent component analysis of CA1 LFPs

To dissociate pathway-specific current generators in CA1 and DG, we applied one-dimensional independent component analysis (ICA) to LFPs (Fernández-Ruiz and Herreras, 2013). LFPs were band-pass filtered between 20 and 200 Hz with a third-order Butterworth filter (butter, zero-phase filtfilt), subtracting the per-channel mean. The filtered data matrix X (channels x timepoints) was then used to compute the covariance matrix.


$$S=\frac{1}{T}XX^{T}$$

Where *T* is the number of samples. We applied the JADE algorithm (Cardoso, 1999) to X, requesting ten independent components. To visualize each component’s spatial distribution, we computed the forward (mixing) model.


$$W_{\mathrm{fm}}\;\;=\mathrm{BS}$$

So that each row of W_fm_ gives the weight of that component at each channel within a shank. To ensure a consistent polarity, we identified each component’s peak channel index and flipped its sign if necessary, so that the maximal absolute weight was positive.

### Unsupervised classification of CSD event clusters

To objectively classify distinct ripples and DSs based on their underlying synaptic current patterns, we performed an unsupervised clustering analysis on the CSD profiles of all detected events.

First, a feature vector was constructed for each event. This vector represented the spatial CSD profile at the time of the event peak, created by concatenating the CSD values across all channels on the selected shank(s). To ensure the classification was based on the spatial configuration of current sinks and sources rather than event amplitude, each event’s CSD vector (Ci) was normalized by its L2-norm:


$${\hat{C}}_{i}=\frac{C_{i}}{{\left\|C_{i}\right\|}_{2}}$$

Where ∥C_i_∥_2_ is the Euclidean norm of the vector. The resulting collection of normalized CSD vectors was organized into a matrix where each row represented an event, and this matrix was subsequently z-scored column-wise prior to further analysis.

Principal component analysis (PCA; *pca* function in MATLAB) was then used for dimensionality reduction. The scores of the first principal component, which captured the largest variance in CSD profile shape, were then used for clustering of ripples. For dentate spikes, the minimum number of PCs needed to capture at least 50% of the total variance was used for dimensionality reduction.

To determine the optimal number of event clusters present in the data, we used a clustering approach based on Gaussian Mixture Models (GMM; *fitgmdist* function in MATLAB). We fitted separate GMMs to the principal component scores, systematically testing a range of possible cluster numbers (*k* = 1 to 10). The optimal model, and thus the most likely number of distinct event types, was selected as the model that yielded the minimum Bayesian Information Criterion (BIC). The BIC provides a principled trade-off between model fit and complexity, penalizing models with a greater number of parameters, and is calculated as:


$$\mathrm{BIC}=\mathrm{pln}(n)-\mathrm{2ln}(\hat{L})$$

Where p is the number of parameters in the model, n is the number of events, and $\hat{L}$ is the maximized value of the model’s likelihood function.

Each event was assigned to a cluster by passing the fitted GMM and data matrix, containing events (rows) x scores for the selected PCs (columns), as inputs to the *cluster* function (Statistics and Machine Learning Toolbox in MATLAB). The *cluster* function then computed the posterior probability of an event belonging to each of the Gaussian distributions in the fitted model, using the mixing proportion learned by the GMM for each cluster as the prior probability. Finally, each event was assigned to the single cluster that yielded the maximum posterior probability.

### Single unit analysis

A concatenated signal file was prepared by merging all recordings from a single animal from a single day. Putative single units were first sorted using Kilosort 2.5 (Pachitariu et al., 2023) which used drift-correction to change templates continuously as a function of drift, and then manually curated the data using Phy2.^2^

### Cell type classification

Cells were classified into three putative cell types: narrow interneurons, wide interneurons, and pyramidal cells. Interneurons were selected by 2 separate criteria; narrow interneurons were assigned if the waveform trough-to-peak latency was less than 0.425 ms. A wide interneuron was assigned if the waveform trough-to-peak latency was more than 0.425 ms and the rise time of the autocorrelation histogram was more than 6 ms. The remaining cells were assigned as pyramidal cells. Autocorrelation histograms were fitted with a triple exponential equation to supplement the classical, waveform feature-based single unit classification^3^ (Petersen et al., 2021). Bursts were defined as groups of spikes with interspike intervals <9 ms.

## Approach, results, and discussion

### Anchoring mesoscopic patterns to anatomical landmarks

**Figure 1:**
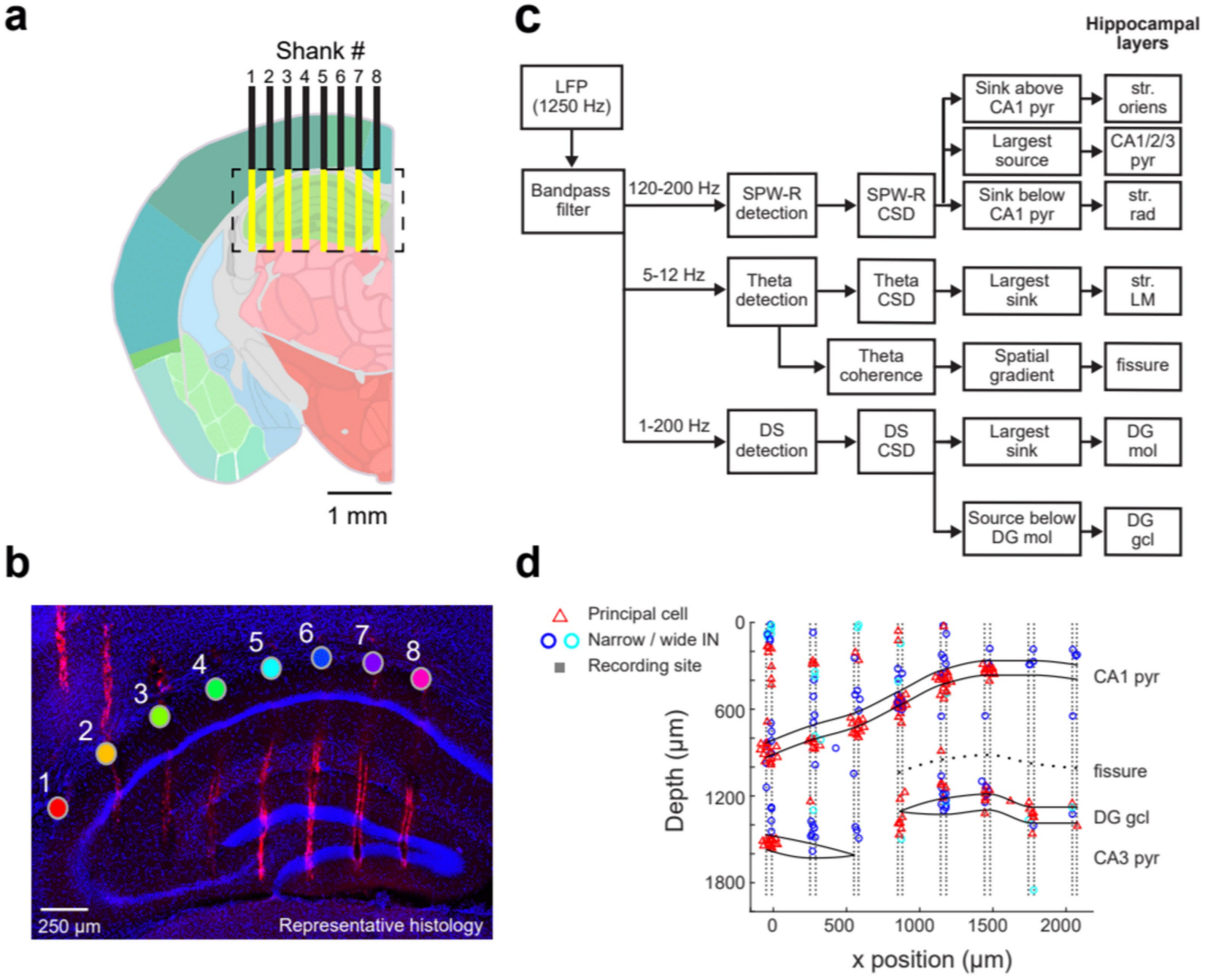
Anatomical coverage and identification of dorsal hippocampal layers. **(a)** Schematic of the ideal hippocampal recording locations (yellow) obtained with the 8-shank SiNAPS probe, spanning 2,188 μm x 1924 μm (width x depth). The probe was implanted 2 mm posterior to Bregma. Shanks are overlaid on the Allen Mouse Brain Atlas (image 73). **(b)** Representative histology of the probe tracks with 4′,6-diamidino-2-phenylindole (DAPI; blue) and DiI (red) staining on a coronal mouse hippocampal section. The filled circles represent the str. Oriens site on each shank, with the shank numbers shown above. The scale bar is 250 μm. **(c)** Workflow for hippocampal layer identification based on current source density analysis and spatial coherence maps of the ripple, DS, and theta frequency bands. **(d)** Putative single units—classified as principal cells, narrow interneurons, and wide interneurons—are plotted at the sites of their spike waveform amplitude maxima. The relative positions of each shank are shown schematically with the cell layer outlines superimposed, defined by the electrophysiological features indicated in **(c)**. The first recording site on each shank corresponds to 0 μm, while the largest depth is at the probe tip. The single-unit data in **(d)** were collected from a different mouse than the histology shown in **(b)**; the latter was obtained shortly after implantation to demonstrate the anatomical coverage of the SiNAPS probe.

Revealing the current sources and sinks of the various hippocampal population patterns requires electrodes with high spatial resolution and large spatial coverage. To achieve this goal, we employed various silicon probe configurations. The SiNAPS probe spans an approximate square area (4.12 mm^2^) distributed across 8 shanks (inter-shank distance of 300 μm), with each shank having two parallel columns of 64 sites (14 × 14 μm with 30 μm inter-electrode distance; Angotzi et al., 2019, 2025). Neuropixels 2.0, with 5,120 recording sites distributed over four shanks, can record from 384 user-selected channels. Channels can be distributed across 4 shanks (inter-shank distance of 250 μm), with each shank having one column of 96 sites (12 × 12 μm with 15 μm inter-electrode distance). Implanting either one SiNAPS or two Neuropixels 2.0 probes can cover almost the entire circuitry of a lamella in the dorsal hippocampus of a mouse (Figures 1a,b; Supplementary Figure S5).

The primary goal of each experiment was to establish reliable correlations between electrical signals and anatomical structure. The high-density 1,024-channel recordings across 8 shanks allowed the decomposition of the LFP signal into its sources. The relationship between afferents and dendrites, as well as somatic layers, and the characteristic depth profiles of various oscillatory LFP patterns, were used to identify hippocampal layers and their transitions (Figure 1c; Supplementary Figure S1; Andersen et al., 1973; Amaral and Witter, 1989; Witter et al., 1989; Belluscio et al., 2012; Colgin, 2016; Schomburg et al., 2014). Because head-fixed recordings allowed data collection for a limited time only, we supplemented the two-dimensional recording experiments with 4-shank Neuropixels recordings and single-shank silicon probe recordings in freely moving mice. The long recordings in these animals allowed us to study electrical patterns during natural sleep.

**Figure 2:**
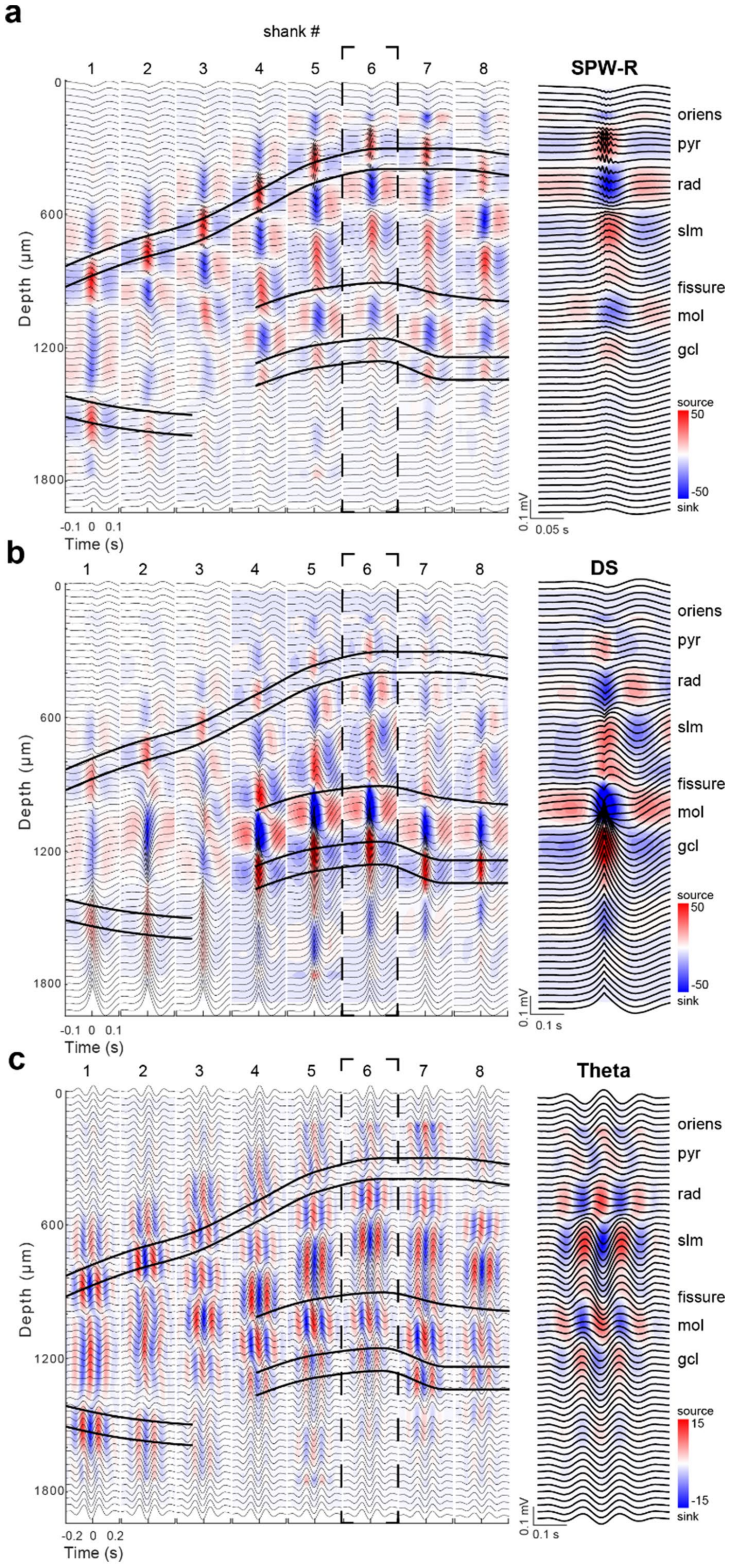
Dorsal hippocampal SPW-R, DS, and theta CSD maps. **(a)** Average SPW-R-triggered CSD profile for events (*n* = 1,164) detected on the CA1 pyramidal layer reference channel on shank 6. The strong current source underlying ripples across each shank was used to identify the pyramidal cell layer of the CA1, CA2, and CA3 subfields. The prominent current sinks (blue) above and below the str. Pyramidale correspond to excitatory inputs arriving from CA2/CA3a (str. oriens) and CA3b-c (str. Radiatum; sharp wave), respectively. Average LFP traces (±100 ms; black) are plotted across depths and overlaid on the CSD plots for each shank. **(b)** Average DS-triggered CSD profile for events (*n* = 1,464) detected in the granule cell layer reference channel on shank 6. The large current sink identifies the dentate molecular layer, corresponding to entorhinal excitation. The coupled deeper source, reflecting feed-forward perisomatic active outward and passive return currents, identifies the granule cell layer. **(c)** Average 2D CSD profile across shanks centered on theta peaks during walking epochs. The theta band-filtered LFP (5–12 Hz) of the str. Oriens reference site on shank 6 was used for theta peak detection. Note the same time and color scales are used for SPW-Rs and DSs, while different scales are applied for theta. Right: Expanded panels of the CSD profiles on shank 6 for each event type in **(a–c)**.

We first analyzed sharp wave-ripples (SPW-Rs) in the pyramidal layer of CA1 during rest/immobility and constructed a ripple-triggered average current source density (CSD) map (Figure 2a). The recording sites with the largest amplitude ripple wavelet in SPW-R events marked the middle of the CA1 pyramidal layer (Mizuseki et al., 2011). This depth corresponded to the strongest current source in the SPW-R-triggered CSD, reflecting outward currents from perisomatic inhibition and passive return currents (English et al., 2014; Hulse et al., 2016; Stark et al., 2014). Similarly, the CA3 pyramidal layer was localized by a current source time-locked to the average SPW-R peak, detected in the CA1 pyramidal channel. A prominent current sink below the CA3-CA1 pyramidal layer denoted the CA3-CA1 str. Radiatum, corresponding to recurrent collateral and associational afferent excitation from CA3 pyramidal cells. In the dentate gyrus, LFP dentate spikes (DS), identified by a sink in the mid-molecular layer, reflected excitatory input from the medial entorhinal cortex (Figure 2b; Bragin et al., 1995b; Dvorak et al., 2021; GoodSmith et al., 2019). The sink-source reversal of the average DS marked the top of the granule cell layer (Senzai and Buzsáki, 2017; Santiago et al., 2024; McHugh et al., 2024). The density of neuronal spiking provided further support for the correct identification of the cell body layers (Figure 1d).

For theta maps, we used averages of filtered theta waves, referenced to theta peaks recorded in the distal CA1 str. Oriens on shank 6 (Figure 2c). The phase of theta waves was uniform above the pyramidal layer and began to shift below the pyramidal layer, reaching a full phase reversal in the CA1 str. Lacunosum-moleculare (180° phase difference). A second prominent theta dipole was localized in the dentate molecular layer, where the strongest sinks corresponded to excitatory inputs from entorhinal layer 2, complementing the inputs from layer 3 to CA1 str. Lacunosum-moleculare (Brankack et al., 1993; Zutshi et al., 2022). In the CA3 region, the interpretation of current sinks and sources is less straightforward due to the structure’s curvature. While the spatial positions of the sinks and sources during theta oscillations are determined by the laminar inputs of excitation (in dendritic layers) and inhibition (mostly in cell body layers), respectively, the magnitude of the sink-source distributions and their temporal relationship can vary as a function of (1) movement types (e.g., paw manipulation vs. running) (Vanderwolf, 1969; Buzsáki et al., 1985); (2) running speed (Buzsáki et al., 1979; Buzsáki et al., 1985; Czurkó et al., 2011; McNaughton et al., 1983); (3) cognitive factors (Schomburg et al., 2014); and (4) REM sleep (Montgomery et al., 2008, 2009).

**Figure 3:**
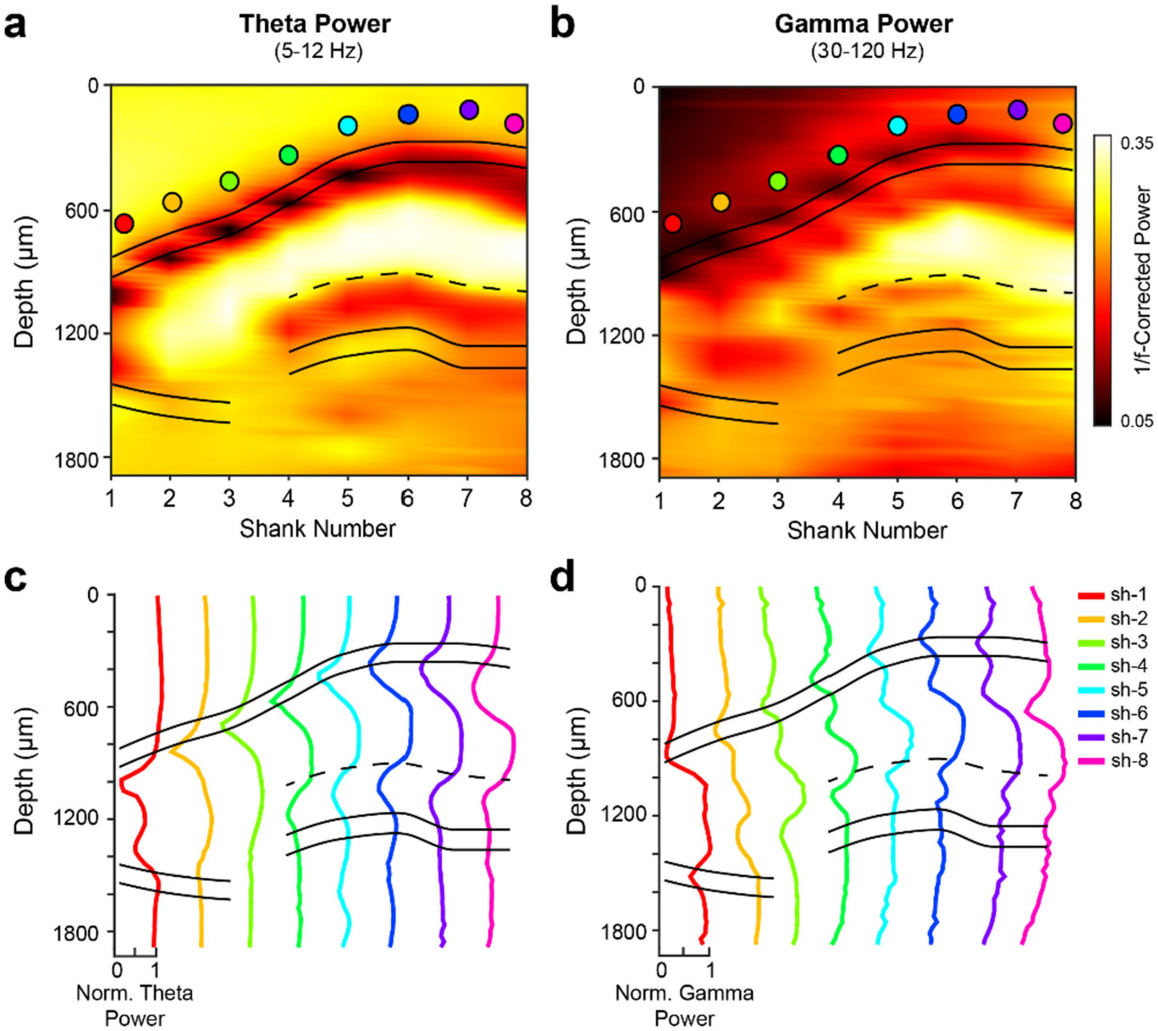
Dorsal hippocampal theta and gamma power maps. **(a,b)** 1/f-corrected theta (5–12 Hz) **(a)** and gamma (30–120 Hz) **(b)** power across the transverse axis of the dorsal hippocampus. Power in each frequency band was computed during wake epochs using Welch’s method. The filled circles represent the str. Oriens reference channel used from each shank; the same color scheme is used in panels **c,d**. The 1/f-correction was applied using the FOOOF toolbox (Donoghue et al., 2020) to account for aperiodic, or direct current, shifts in the power spectrum profiles of the broadband LFP across hippocampal layers. **(c,d)** Within-shank depth profiles demonstrating relative changes in theta **(c)** and gamma **(d)** 1/f-corrected power values. Data were normalized to the highest value across shanks in each respective frequency band.

Theta phase shifted not only in the CA1-dentate axis, but also in the medio-lateral direction (Lubenov and Siapas, 2009; Oliva et al., 2016a). To quantify the magnitude of the phase shift in the transverse axis, we chose the most medial (distal CA1) or most lateral (at the proximal CA1-CA2 border) site in the stratum (str.) oriens as reference and computed a global theta phase map (Supplementary Figure S3). Theta phase maps with reference channels corresponding to distinct hippocampal layers on each shank were also computed (Supplementary Figure S3). The phase shift between proximal CA3 and distal CA1, measured in the str. Oriens, corresponded to approximately 180^o^, irrespective of the reference site, which mainly affected the amplitudes of the wave at different locations. While 1/f-corrected theta power showed a relatively uniform distribution in the medio-lateral axis (Figure 3a; Supplementary Figure S2), theta waves emerge medially and propagate laterally (Supplementary Movie S1), in addition to the traveling theta waves along the long axis of the hippocampus (Lubenov and Siapas, 2009; Patel et al., 2012). These power and phase depth profiles are compatible with decades of research on hippocampal theta oscillations (Berényi et al., 2014; Bragin et al., 1995a; Brankack et al., 1993; Buzsáki, 2002; Buzsáki et al., 1986; Lubenov and Siapas, 2009; Sullivan et al., 2014; Winson, 1974).

**Figure 4:**
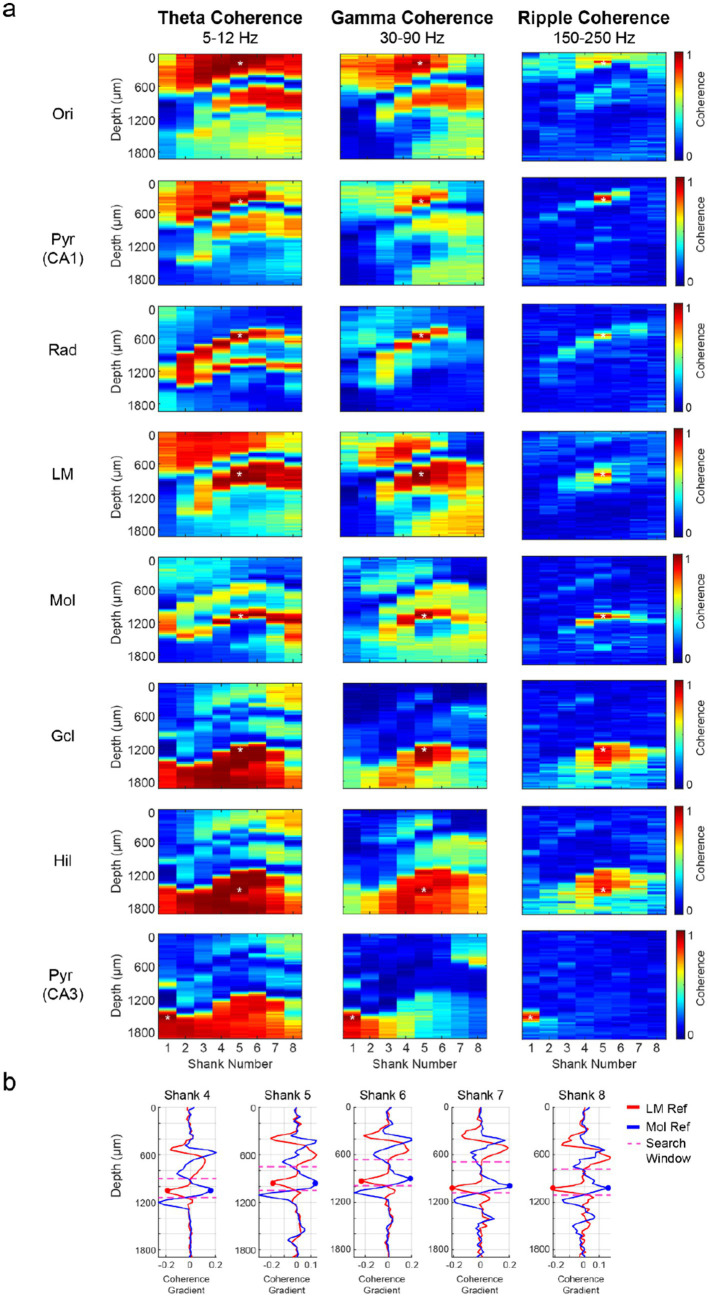
Localization of the hippocampal fissure by coherence maps. **(a)** The LFP from reference channels (asterisk) across hippocampal subregions was filtered in the theta (5–12 Hz), gamma (30–90 Hz), and ripple (150–250 Hz) frequency bands. The magnitude-squared coherence was computed using 300 non-overlapping 1-s intervals from wake and NREM sleep epochs for the theta/gamma and ripple coherence maps, respectively. Note the high coherence within layers and rapidly decreasing coherence across layers. **(b)** Spatial coherence gradients were computed for shanks 4–8 to identify the hippocampal fissure. The spatial coherence gradient indicates the change in coherence between neighboring channels at different depths, as filtered by theta (5–12 Hz), gamma (30–90 Hz), and ripple (150–250 Hz) frequency bands. Coherence values at each site reflect the average between adjacent channels from the same shank and depth (28 μm horizontal spacing). Str. lacunosum-moleculare (Slm) and dentate molecular layer (Mol) reference channels from each shank were used to restrict the search window (dotted lines) for fissure detection. The depth corresponding to a negative change in the Slm-referenced plot and a positive change in the Mol-referenced plot indicated the anatomical boundary between these layers and thus the location of the hippocampal fissure. Pyr: pyramidal cell layer; Rad: str. Radiatum; Gcl: dentate granule cell layer.

The hippocampal fissure, which separates the CA1 and dentate regions, was initially estimated by measuring the distance between the CA1 str. Pyramidale and dentate granule cell layer across the medial-lateral axis in histological sections (Supplementary Figure S4). The ratio between the distance of the CA1 str. Pyramidale to the fissure and the distance of the fissure to the dentate granule cell layer was approximately 2:1. This ratio systematically increased from the lateral edge of the hippocampal fissure to the medial border of the dorsal hippocampus (1.53 to 2.79). A more precise approach for identifying the CA1-dentate boundary was based on theta and gamma coherence maps, distinguishing the str. Lacunosum-moleculare from the dentate molecular layer by the asynchronous inputs to these subregions during awake epochs (Figure 4a; Berényi et al., 2014). As expected, higher frequencies (gamma: 30–90 Hz; ripple: 150–250 Hz) displayed more localized coherence compared to the lower frequency (theta: 5–12 Hz) coherence map (Figure 4a).

The CA1 str. Lacunosum-moleculare (Slm) and dentate molecular layer (Mol)-referenced coherence maps were used to compute spatial coherence gradients separately in each shank. A search window was specified between the Slm and Mol reference channels to identify the fissure. A local trough in the Slm-coherence gradient plot, corresponding to the lower boundary of CA1, and a local peak in the Mol-coherence gradient plot, marking the upper boundary of the dentate gyrus, marked the hippocampal fissure location (Figure 4b). The transition points in the coherence gradient plots for each shank generally converged onto the same depth, although rarely these points were offset by one channel (~30 um). In this case, the fissure depth was approximated as the average depth of both transition points. The coherence-based identification of the hippocampal fissure yielded similar results regardless of the sublayer-specific reference site chosen or whether theta or gamma coherence was used (Figure 4).

Mice with two 4-shank Neuropixels (Supplementary Figure S5) and single shank linear probe recordings (Zutshi et al., 2022) also enabled sampling of LFP in the dorsal hippocampus, at least in the dorso-ventral axis. In support of the head-fixed observations with the 1,024 channel SiNAPS probe, theta waves in CA1 showed a gradual phase shift from the str. Oriens to str. Lacunosum-moleculare with an amplitude dip just below the CA1 pyramidal layer. In addition, the prominent theta sink in the dentate molecular layer was approximately 180^o^ phase-shifted relative to the sink in CA1 lacunosum-moleculare (Zutshi et al., 2022). The sink-source pair in the dentate molecular layer and the hilus was also reflected by the phase-reversal of theta waves across the granule cell layer, including in the inner blade of the dentate gyrus. The 180^o^ phase shift between theta sinks in CA1 str. Lacunosum-moleculare and dentate molecular layer reflects the theta phase-reversed preferred firing of layer 2 and layer 3 entorhinal cortical neurons (Mizuseki et al., 2009).

### Theta-gamma relationship

Gamma oscillations occupy a broadband pattern from ~30 Hz to ~120 Hz, with the exact sub-bands varying as a function of brain region, sublayer, and behavior (Buzsáki and Wang, 2012). Thus, they have a variety of forms, including (1) locally generated patterns, particularly in the pyramidal layers; (2) projected patterns, mainly from the CA3 (low gamma; 25–55 Hz) and entorhinal cortex (high gamma; 60–100 Hz); and (3) passive return currents (Colgin, 2015; Lopes-dos-Santos et al., 2025; Schomburg et al., 2014; Trimper et al., 2017).

Extraction of gamma oscillation features requires several cautionary steps. Simply looking at the power in a particular band is misleading because it is superimposed on the wideband power. Moreover, there is a large, systematic shift in the broadband power spectrum across hippocampal layers. Thus, gamma power depth profiles measured directly from the power spectrum are very similar to theta depth profiles (Figures 3c,d) (Supplementary Figures S2a,b). An important step is to remove the 1/f wideband pink noise (Supplementary Figures S2e,f) (Figure 3b). After such correction, distinct gamma patterns emerge, including local peaks in the dendritic layers (rad, slm, and mol; Figures 3b,d). Another caution is that the second and third harmonics of theta power are very prominent during running (Buzsáki et al., 1985), leading to spurious low “gamma” oscillations, particularly in the CA1 str. Radiatum, a major target of CA3 output (Sheremet et al., 2019a; Sheremet et al., 2019b; Qin et al., 2023). Separation of harmonics from true gamma oscillations requires further analytical and perturbation experiments (Lopes-dos-Santos et al., 2018).

**Figure 5:**
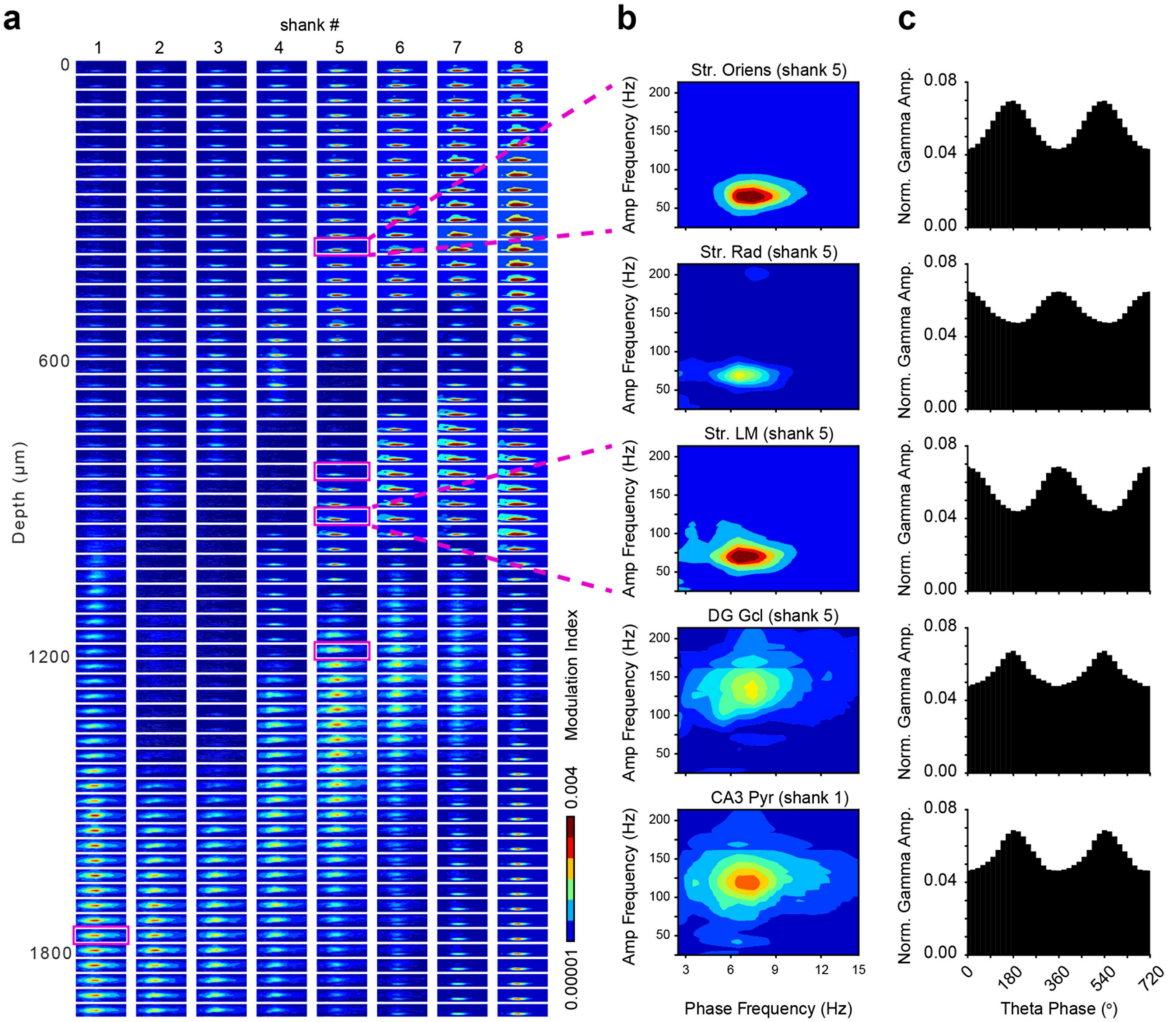
Phase-amplitude coupling of theta and gamma rhythms in the dorsal hippocampus. **(a)** Spatial map of cross-frequency coupling (CFC) strength across 512 channels of the SiNAPS probe, using one vertical column of channels from each shank. Each subplot represents a comodulogram for a single recording site, plotting the Modulation Index (MI) as a function of phase frequency (2–15 Hz) and amplitude frequency (25–220 Hz). The color indicates the MI strength. **(b)** Representative comodulograms from five anatomically distinct regions (CA1 str. Oriens, CA1 str. Radiatum, CA1 str. Lacunosum-moleculare, DG molecular layer, and CA3 str. pyramidale), corresponding to the magenta boxes on the left panel. **(c)** Corresponding phase-amplitude histograms showing normalized gamma amplitudes locked to the phase of the local theta rhythm (two cycles shown, 0–720 degrees). The MI was calculated using a Kullback–Leibler divergence-based metric to quantify the deviation of the empirical amplitude distribution from a uniform distribution.

Theta-gamma phase-amplitude coupling (PAC) is evident in all subregions of the hippocampus. However, the specific coupled frequencies vary across hippocampal regions and layers, as well as the behavioral task and cognitive demands. For example, the theta-modulated gamma band has been shown to occur at a higher frequency in the DG (80–120 Hz) compared to the str. Radiatum (40–70 Hz) (Colgin et al., 2009; Schomburg et al., 2014; Buzsáki and Schomburg, 2015). We computed a spatial map of the cross-frequency coupling (CFC) strength across regions of the dorsal hippocampus to expand on previously reported patterns with higher resolution recordings (Figures 5a,b). Theta-modulated gamma power was maximum near the peak of the local CA1 theta oscillation recorded from the apical dendritic layers (str. Radiatum and str. Lacunosum-moleculare), whereas in the basal dendritic layer (str. oriens), theta-modulated gamma power of the same frequency range (40–70 Hz) was largest at the theta trough (Figure 5c). In the DG granule cell layer and CA3 pyramidal layer, higher frequency gamma (80–140 Hz) exhibited the strongest cross-frequency coupling to local theta, measured by the Modulation Index (Figure 5c; Tort et al., 2010). Numerous factors, such as attention, learning, memory, spatial context, motor behavior patterns and brain state can affect theta-gamma phase-amplitude coupling. An often overlooked but prominent modulator of this relationship is respiration, which can induce transient coupling-decoupling (Karalis and Sirota, 2022; Tort et al., 2025).

### Types of sharp wave-ripples (SPW-Rs)

The genesis and physiological functions of SPW-Rs have been the subject of numerous studies conducted on various species, ranging from rodents to humans (Buzsáki, 2015; Buzsáki and Fernández-Ruiz, 2019; Colgin, 2016; Dickey et al., 2022; Girardeau and Zugaro, 2011; Iwata et al., 2024; Jadhav et al., 2012; Joo and Frank, 2018; Liu et al., 2022; Schlingloff et al., 2014; Tingley et al., 2021; Yang et al., 2024). SPW-Rs are induced by intrahippocampal dynamics. When the levels of subcortical neuromodulators decrease to a minimum, it allows the excitatory recurrent collaterals of CA2-3 pyramidal neurons to induce a population burst. These population bursts are also observed in isolated hippocampal slices *in vitro* and in transplanted hippocampal tissue (Bazelot et al., 2016; Buzsàki et al., 1987; Traub, 1982), supporting the intrahippocampal genesis of SPW-Rs. In the intact hippocampus, the CA3 population burst depolarizes the apical dendrites of CA1 pyramidal neurons, detected extracellularly as a sharp wave (SPW) in the mid-str. Radiatum (Buzsáki et al., 1983). When, occasionally, the CA2 neurons initiate a population burst, a negative SPW (sink) is present in the CA1 str. Oriens, reflecting the excitation of the basal dendrites of CA1 pyramidal cells (Oliva et al., 2016b). In both cases, the synchronous excitation activates perisomatic interneurons (mainly basket and bistratified cells; Klausberger and Somogyi, 2008), and their fast interactions produce a fast oscillation, i.e., the ripple (Figure 2a; Buzsáki et al., 1992; Stark et al., 2014). The extracellularly recorded ripple is a combination of superimposed spikes (negative part; Schomburg et al., 2012) and the fast IPSCs between spikes in pyramidal neurons (positive part; English et al., 2014) Below, we focus on recently discussed aspects of SPW-Rs.

Different types of SPW-Rs have been suggested (Aleman-Zapata et al., 2025; Chenani et al., 2019; Liu et al., 2023; Nakashiba et al., 2009; Nakashiba et al., 2008). The first distinction is waking/resting SPW-Rs and SPW-Rs during NREM sleep, with ripples 10–20 Hz faster during NREM than in the waking animal (Petersen et al., 2022; Roumis and Frank, 2015; Jadhav et al., 2016). SPW-Rs also have longer duration in novel environments and when the hippocampus is involved in the task (Fernández-Ruiz et al., 2019). Within the same brain state, waveform clustering methods identified distinct putative SPW-R types from single-site recording and functional imaging (Logothetis et al., 2012; Sebastian et al., 2023). Another classification scheme uses the temporal relationship between SPW-Rs and neocortical UP-DOWN states of slow oscillations because the neocortex and hippocampus are bidirectionally connected. During the active (UP) state of the neocortex, many more SPW-Rs occur than during cortical silent (DOWN) states. However, the majority of SPW-Rs either precede the neocortical U-D transition (SPW-R_UD_) or follow D-U transitions (SPW-R_DU_). SPW-Rs during cortical DOWN states have the smallest amplitude. Large-amplitude SPW-Rs are followed by an increased probability of DOWN states in the retrosplenial cortex and the spreading of the DOWN state along cortico-cortical connectivity. In the reverse direction, sharp D-U transitions in the default mode network (known as K-complexes; Amzica and Steriade, 1997; Colrain, 2005) can induce hippocampal SPW-Rs with a characteristic latency, illustrating that weakly coupled chaotic systems can effectively bias the timing of events in partner structures (Swanson et al., 2025). The relationship between the U-D-related SPW-R types and those based on waveform analysis needs to be elucidated in future research.

The above classic picture of intrahippocampal genesis of SPW-Rs was recently challenged, suggesting that CA1 ripples can also be induced or modified by extrahippocampal input (Nakashiba et al., 2009; Castelli et al., 2025). In principle, any strong depolarization, including optogenetic activation of interneurons, can induce a ripple not only in CA1 but also in CA3, dentate gyrus, and even cortical regions (Stark et al., 2014). In addition, theta oscillations can occasionally induce fast gamma oscillations, which overlap with the ripple band (Buzsáki, 2015). Castelli et al. (2025) described two ripple types based on their distinct laminar currents across the CA1 somato-dendritic axis. In addition to the canonical SPW-R with a strong sink in str. Radiatum (‘Rad^sink^’ ripples; 147 Hz), they observed fast oscillations in the CA1 pyramidal layer associated with a current sink in CA1 str. Lacunosum-moleculare (‘LM^sink^’ ripples; 125 Hz). Using a linear discriminant classifier, trained on CA1 pyramidal layer LFP waveforms, the classifier distinguished putative Rad^sink^ and LM^sink^ events. In turn, the authors applied the classifier results to tetrode recordings in CA1 and characterized other features of these putative SPW-R events. Rad^sink^ ripples were coincident with increased CA3 pyramidal neuron activation. They recruited both superficial and deep-layer principal cells, integrating them into higher-dimensional composite patterns that reactivated recently expressed waking motifs of neuronal coactivity, consistent with previous work (Valero and De La Prida, 2018). In contrast, LM^sink^ ripples recruited mainly motifs from deep-layer CA1 pyramidal cells, which formed a lower-dimensional pattern that underwent reactivation drift during post-learning rest or sleep. CA3 pyramidal neurons fired mainly *after* the LM^sink^ ripple. These new findings suggest that current sinks in CA1 str. Lacunosum-moleculare can also induce bona fide SPW-Rs.

**Figure 6:**
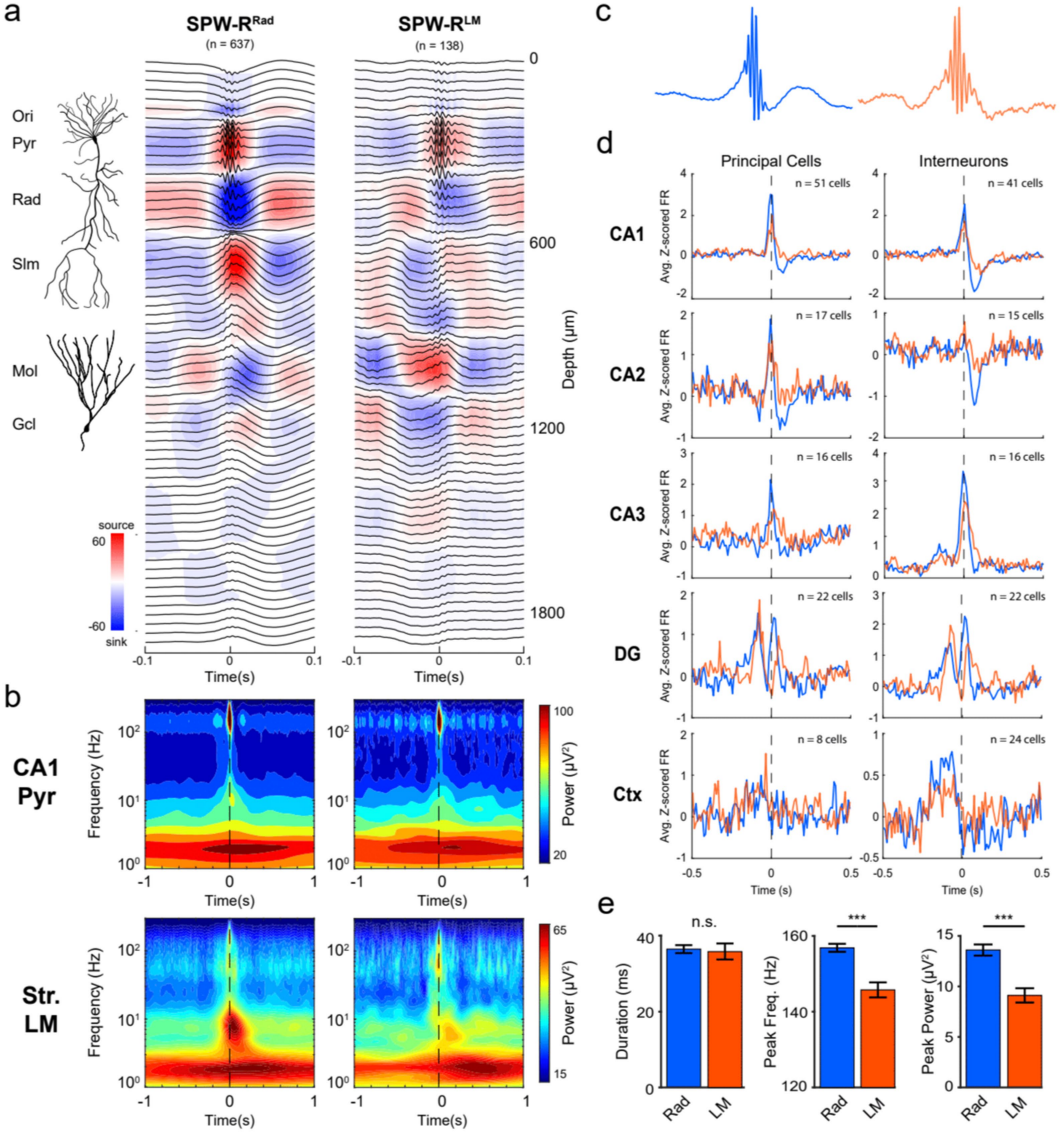
SPW-R subtypes based on CSD profiles. **(a)** Average SPW-R-triggered (± 0.1 s) CSD profiles. For all SPW-Rs detected on the CA1 pyramidal channel on shank 6 (1,164 events), CSD values across depths at the event peak were vectorized and used as input for PCA. The number of meaningful clusters present was determined by selecting the GMM with the lowest BIC value. Using only the 1st PC resulted in SPW-Rs with a strong current sink in the str. Radiatum (SPW-R^Rad^; *n* = 637) and a separate group with a prominent current sink in the str. Lacunosum-moleculare at the event peak (SPW-R^LM^; *n* = 138). Note that a third cluster with coincident sinks in both the str. Radiatum and mid-molecular layer of the DG (SPW-R^DS^; *n* = 389) is not shown here due to its similarity in LFP features and neuronal spiking with the SPW-R^Rad^ cluster (Supplementary Figures S6–S8). An illustration of a reconstructed CA1 pyramidal cell and dentate granule cell is included. **(b)** For each SPW-R subtype, the average broadband spectrograms (1–300 Hz) are shown for the CA1 pyramidal (top) and str. Lacunosum-moleculare (LM; bottom) channels. Frequencies are plotted in log-scale to visualize the low-frequency embedding of each cluster. **(c)** Average CA1 pyramidal channel LFP centered on each SPW-R subtype (±0.25 s). **(d)** Neuronal spiking correlations for each SPW-R cluster. The average z-scored principal cell (left) and interneuron (right) firing rate surrounding SPW-R^Rad^ (blue) and SPW-R^LM^ (orange) shown for all recorded regions (CA1, CA2, CA3, DG, and Ctx). **(e)** LFP features for each SPW-R subtype. The peak frequency and ripple power of the SPW-R^LM^ were significantly lower compared to the SPW-R^Rad^. Asterisks indicate *p* < 0.0001; n.s. indicates no significant difference between groups.

Using our high-density electrodes, we classified SPW-Rs by principal component analysis (PCA). The first three principal components accounted for >55% of the variability (Supplementary Figure S6). Using only the 1st principal components resulted in potentially three different SPW-Rs with (1) a prominent current sink in the str. Radiatum (SPW-R^Rad^); (2) large current sinks in both the str. Radiatum and dentate molecular layer (SPW-R^DS^); and (3) a current sink in the str. Lacunosum-moleculare at the event peak, flanked by sources in str. Radiatum and the dentate molecular layer (SPW-R^LM^; Figure 6). By this classification, (SPW-R^DS^) corresponds to the classical type. SPW-R^DS^ is virtually identical to SPW-R^Rad^ but it coincides with a DS (Supplementary Figure S7) in the dentate molecular layer. SPW-R^LM^ corresponds to the LM^sink^ ripples described by Castelli et al. (2025).

SPW-R^LM^ differed from the other types in multiple aspects. First, its ripple frequency was slower (145.7 Hz, 95% CI [143.7–147.7]) compared to SPW-R^Rad^ (156.9 Hz, 95% CI [155.8–158.0]) and SPW-R^DS^ (154.3 Hz, 95% CI [152.9–155.7]). Second, SPW-R^LM^ was preceded by a large negative wave and corresponding sink in the dentate molecular layer (~ 80 ms before the peak of the CA1 ripple power; Figure 6a). Third, they differed in the relationships to single unit activations in different subregions of the hippocampus (CA1, CA2, CA3, and DG) and neocortex (Figure 6d).

The spike correlations of the two types of SPW-R^Rad^ and SPW-R^DS^ were similar, although with stronger spike discharges of CA3-CA1 neurons during SPW-R^Rad^ and stronger spike discharges of dentate gyrus neurons during SPW-R^DS^ (Supplementary Figure S8). Discharges of CA1-CA3 pyramidal neurons during SPW-R^Rad^ and SPW-R^DS^ were followed by suppression of spiking activity. Inhibitory neurons largely followed the temporal dynamics of pyramidal cells. In contrast, SPW-R^LM^ events were associated with weaker excitation of CA1 pyramidal neurons (Figure 6d), as expected from the weaker sinks and sources (Figure 6a). The largest differences were reflected by the reduction (1.22 Hz vs. 2.15 Hz peak z-scored firing rate in SPW-R^LM^ vs. SPW-R^Rad^) and delayed onset of firing of CA3 pyramidal neurons with respect to the ripple peak (+15 ms vs. − 5 ms in SPW-R^LM^ vs. SPW-R^Rad^; Supplementary Figure S8), suggesting that the CA3 recurrent system did not trigger these ripple events (Castelli et al., 2025). Instead, these findings support an extrahippocampal source of drive for SPW-Rs^LM^, possibly driven by layer 3 entorhinal inputs to the apical dendrites of CA1 pyramidal cells. Because SPW-Rs^LM^ were often characterized as repeating events at ~150–250 ms (Supplementary Figure S8b), they may be linked to ~4–5 Hz oscillations, which is the most prominent rhythm in the mouse brain (Einstein et al., 2017; Senzai et al., 2019; Nitzan et al., 2024).

### Generation and taxonomy of dentate spikes (DS)

**Figure 7:**
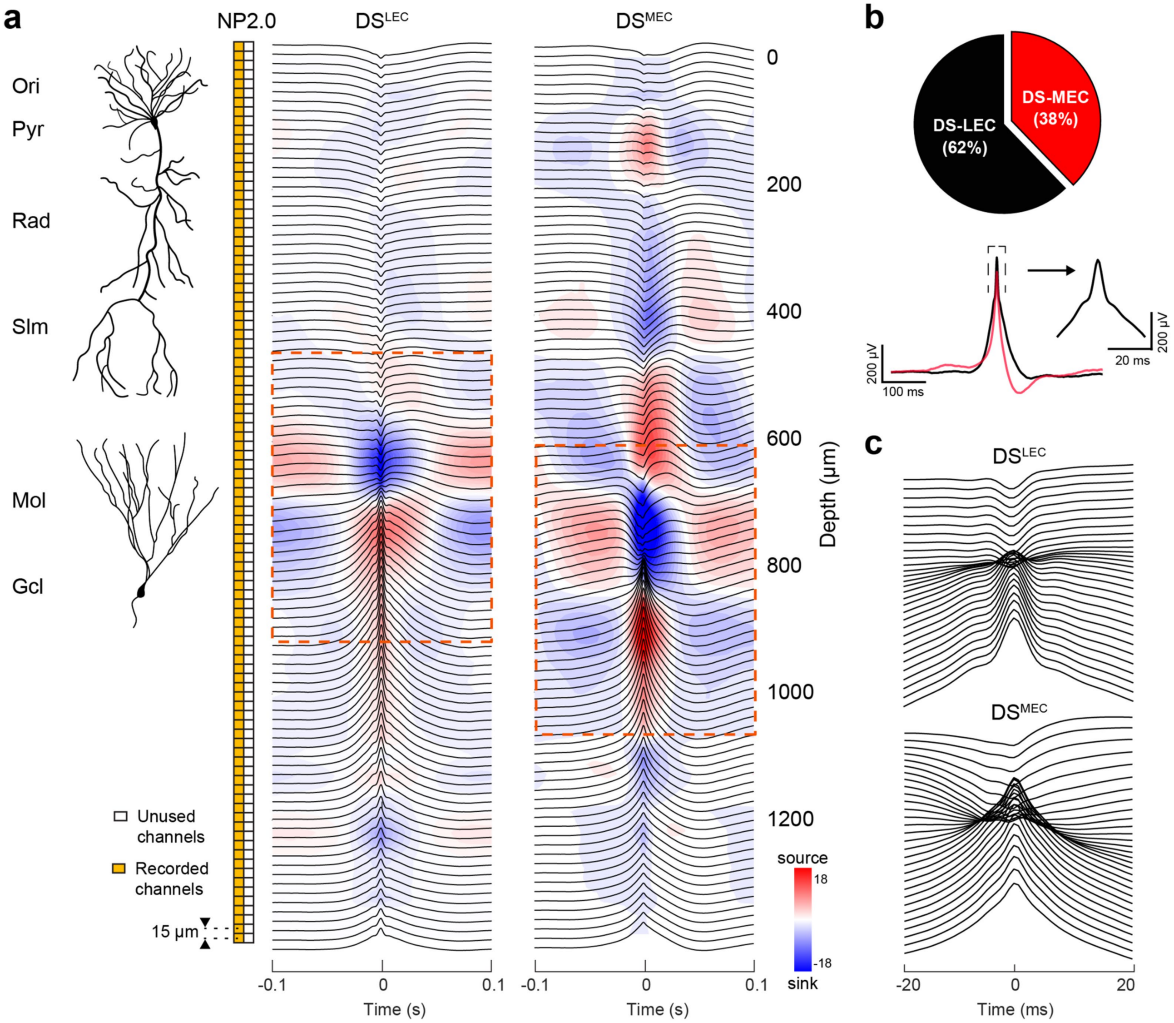
CSD-based classification of dentate spikes triggered by LEC and MEC inputs. **(a)** Laminar Neuropixels 2.0 recording (96-channel, orange boxes) allowed simultaneous monitoring of LFPs across hippocampal layers for CSD analysis (15 ⌠m spacing). Right: Radially organized, mean LFP traces (black) with superimposed CSD profile for DS^LEC^ (5,582 events) and DS^MEC^ (3,392 events). The dominant current sinks in the outer- and mid-molecular layers of the DG are ~100 ⌠m apart (see Supplementary Figure S9). **(b)** Left: average LFP waveform of the DS^LEC^ (62% of total DS events) and DS^MEC^ (38%) referenced to the DG granule cell layer channel used for detection. Note the asymmetry of the DS^MEC^. (Right) Zoomed-in average LFP waveform of the DS^LEC^ around the dashed box in the left. **(c)** Time-expanded panels (±20 ms) for each DS type demonstrating the channels surrounding their respective sink-source phase reversals.

LFP dentate spikes (DS; Figures 1b, 7) represent another intermittent, hypersynchronous population burst pattern in the hippocampus (Bragin et al., 1995b). Although different from SPW-Rs in both neuronal substrates and mechanisms, both patterns are associated with the sequential replay of waking experiences (Farrell et al., 2024; McHugh et al., 2024). Experimentally perturbing DSs or their immediate consequences interferes with memory consolidation (Nokia et al., 2017; Farrell et al., 2024; McHugh et al., 2024).

In the original description of LFP DSs, two types (DS1 and DS2) were distinguished by their differing depth profiles and sink-source distributions (Figure 7; Bragin et al., 1995b). They emerged synchronously and bilaterally in the absence of theta oscillations during waking immobility and non-rapid eye movement (NREM) sleep. DS1 showed a sink in the outer third of the molecular layer, whereas DS2 had a fast sink-source pair with the sink located in the middle third of the molecular layer. DS2 displayed a depth profile identical to the evoked responses to stimulation of the medial entorhinal cortex (MEC; McNaughton and Barnes, 1977; Bragin et al., 1995b) and similar to evoked responses to auditory tone, click, tooth pulp, or air puff sensory stimulation (Brankack and Buzsáki, 1986; Deadwyler et al., 1981; Farrell et al., 2024). These responses likely reflected a startle response mechanism relayed from the brainstem to the entorhinal cortex-hippocampus rather than from the neocortex since the “sensory-evoked DS2” (Farrell et al., 2024) was preserved after removing the sensory cortical areas (Brankack and Buzsáki, 1986). In contrast, the sink of DS1 in the outer molecular layer likely reflected activation from LEC. Surgical removal of the entorhinal input abolished DSs (Bragin et al., 1995b).

In addition to the distinct activation inputs, the two DS types also displayed different morphological properties. While DS1s were detected as isolated, large amplitude fast waves, surrounded symmetrically by gamma waves, DS2 lacked flanking gamma waves, and the LFP spike in the hilus emerged after the suppressed firing of dentate neurons. DSs were associated with large depolarizing potentials in granule cells and occasional spikes (Penttonen et al., 1997). All interneurons examined discharged during DSs (Sik et al., 1997; McHugh et al., 2024), although no distinction was made between DS1 and DS2.

In light of recent observations, the distinction of DS types may need revision. First, the distinct depth distributions of current sinks and sources of DSs clearly correspond to the activation of the dentate circuit by the lateral and medial entorhinal input (LEC and MEC), respectively. Thus, to distinguish between the *origin* of activation, DS_MEC_ and DS_LEC_ designations are more precise and appropriate. The anatomical distinction of the two entorhinal inputs is present not only during DSs but in all brain states. Dvorak et al. (2021) compared the depth profiles of DSs and gamma waves in mice. They demonstrated that MEC and LEC inputs can generate both DSs and gamma oscillations during immobility and running, respectively. This is not surprising because DS1 was initially defined as an isolated large gamma wave (Bragin et al., 1995b). Similarly, during exploration, slow (30 to 50 Hz) and fast (100 to 150 Hz) gamma oscillations are concurrent with theta waves in the rat (Fernández-Ruiz et al., 2021). The low- and high-frequency gamma sub-bands are dominant in the outer (LEC target) and middle third (MEC target) of the dentate molecular layer, respectively, and their amplitude maxima are locked to different phases of theta oscillations. During spatial learning, fast gamma (100–150 Hz) oscillations synchronize the MEC and dentate gyrus, entraining predominantly granule cells. In contrast, during object learning, slow gamma (30 to 50 Hz) oscillations synchronized the LEC and dentate gyrus, preferentially recruiting mossy cells and CA3 pyramidal neurons, indicating task-specific routing of MEC and LEC messages in the form of gamma-cycle-spike packets of selected cell types. These separate communication channels can explain why DS^MEC^ and DS^LEC^ rarely occur together in the same time frame. Overall, these findings demonstrate that distinct gamma-frequency-specific communication between the entorhinal cortex and the dentate gyrus engages functionally related cell assemblies across brain regions in a task-specific manner (Fernández-Ruiz et al., 2021). Recent works have begun to explore the behavioral and cognitive significance of DSs (Nokia et al., 2017; Farrell et al., 2024; McHugh et al., 2024).

McHugh et al. (2024) analyzed DS^MEC^ and DS^LEC^ in resting/sleeping mice and their impact on CA3 and CA1 neuronal populations. They found that DS^MEC^ was more dominant than DS^LEC^ (66% vs. 34%), although this distribution may depend on the nature of the task (Fernández-Ruiz et al., 2021; Santiago et al., 2024). Both DS types activated dentate neurons more strongly compared to CA1 SPW-Rs. DS^MEC^ was more effective in recruiting dentate CA3, and CA1 principal cells compared to DS^LEC^. DSs nested stronger motifs of coactive neurons, yielding population patterns of higher diversity and dimensionality compared with those in SPW-Rs. The offline reactivation of waking population patterns in post-learning rest/sleep DSs contained more diverse and higher-dimensional patterns of neuronal coactivation than those found in SPW-Rs. McHugh et al. (2024) concluded that DSs constitute a second offline network event, in addition to SPW-Rs, that is central to hippocampal population dynamics and supports memory-guided behavior.

Farrell et al. (2024) also distinguished between DS^MEC^ and DS^LEC^ in resting/sleeping head-fixed mice. Similar to SPW-Rs, DSs were associated with brain-wide changes in neuronal activity. Across many brain areas, units firing during DS^MEC^ were often distinct from those discharging during DS^LEC^ and SPW-Rs. DS^LEC^ and SPW-Rs activated similar cortical patterns and suppressed activity in subcortical areas. In contrast, DS^MEC^ activated most cortical and subcortical areas, with high mutual information across brain regions. DS^LEC^ events were less frequent and activated fewer dentate neurons, compared to DS^MEC^, which robustly recruited many cells. Chandelier cells were strongly activated during DS_MEC_, whereas during DS^LEC^ and SPW-Rs they were mainly silent. Brain states into which these three patterns were embedded were also different. Farrell et al. (2024) observed that DS^MEC^ were often present during facial and eye movements, followed by pupil dilation. Moreover, loud noise and air puff stimulation that startled the mouse evoked dentate responses, reminiscent of spontaneous DS^MEC^ (Brankack and Buzsáki, 1986). Finally, the mouse’s current location was replayed by hippocampal neurons during DS^MEC^, in contrast to SPW-R replay, whose spike sequences reflected remote virtual locations of the mouse. The experiments of Farrell et al. (2024) were performed in a head-fixed setting; therefore, the distinction between waking immobility and NREM sleep brain states was not clear. While these experiments provided support for the differences between DS1 and DS2 events, the depth-specific sinks could more generally be interpreted as selective activation of entorhinal afferents from layer III (EC3), which project to CA1 stratum lacunosum-moleculare, and layer II (EC2), which target the dentate gyrus molecular layer. These CSD patterns can represent dentate spikes, theta-related gamma oscillations, or their combinations.

Our current observations are in line with these recent reports (Farrell et al., 2024; McHugh et al., 2024) and extend them in multiple ways. DSs can be classified in at least two different ways. First, by examining their driving (input) sources. Second, they can be classified by their impact on target structures (outputs). Both input and output classifications depend on the instantaneous brain states.

To examine the role of inputs, we used CSD analysis of DSs to derive the canonical two DS types (Figure 7). Candidate events detected from both wake and sleep epochs were first forcibly classified into two clusters based on their CSD profiles at the event peak. One subclass showed a current sink in the outer molecular layer of the dentate gyrus (DS^LEC^), whereas the other was characterized by a sink in the mid-molecular layer (DS^MEC^), corresponding to lateral and medial entorhinal inputs, respectively. In addition to the different sink locations, the two groups differed by waveform, amplitude, and, especially, number of sink-source pairs in the CA1 region. Another distinguishing feature was a prominent ~5 ms sharp peak superimposed on the slower LFP slope in the DS^LEC^ group (Figure 7b, right). In a second approach, we used independent component analysis (ICA) decomposition of LFPs. This analysis yielded three main ICs, with inward currents predominantly restricted to the outer, mid, and inner third of the dentate molecular layer, corresponding to inputs from LEC, MEC, and the mossy cell associational/commissural inputs (Supplementary Figure S9; Lavenex and Amaral, 2000).

**Figure 8:**
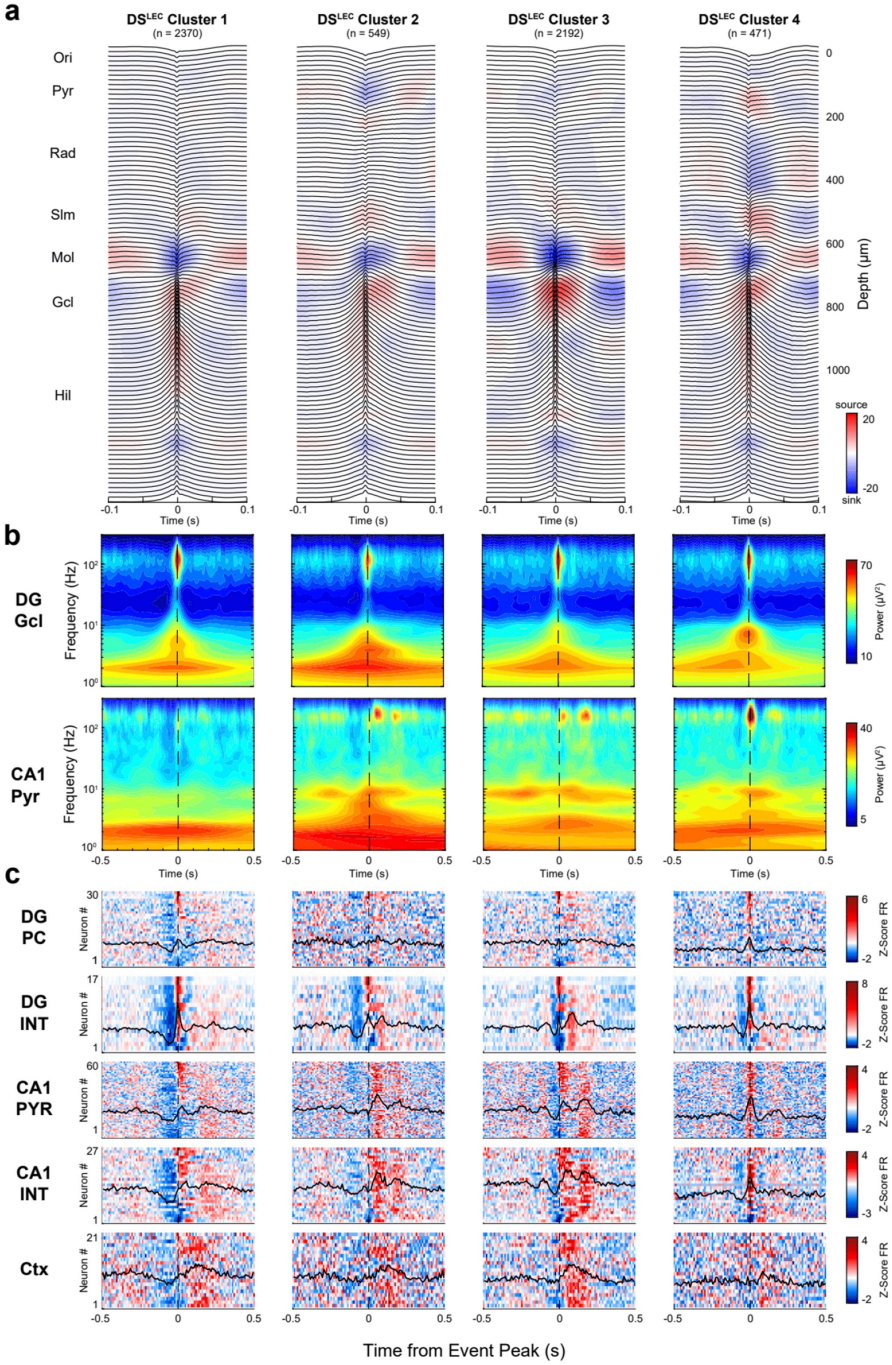
CSD-based DS^LEC^ clusters are distinguished by neuronal spiking patterns. (a) Average LFP traces with superimposed CSD profiles for DS^LEC^ subclusters. Clusters were identified by a GMM following dimensionality reduction with PCs 1–3, accounting for 53% of the total variance. **(b)** Average broadband spectrograms (1–300 Hz) shown with log-scale frequency for each DS^LEC^ subtype referenced to the DG granule cell (top) and CA1 pyramidal channels (bottom). **(c)** Heatmaps show the average z-scored firing rates for DG principal cells (PC), CA1 pyramidal cells (PYR), interneurons (INT), and all cortical neurons (Ctx) recorded simultaneously in a session. For each heatmap: one cell per row, neurons were ranked (top-to-bottom) based on DS^LEC^ cluster 1.

**Figure 9:**
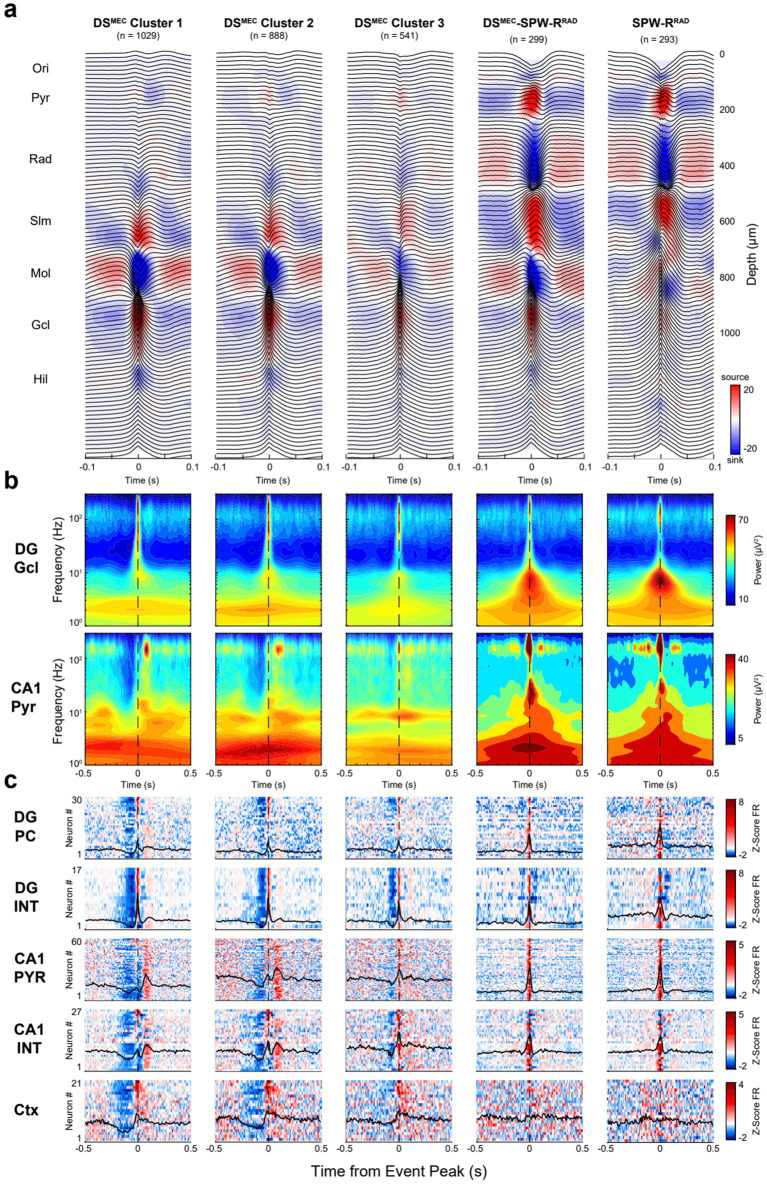
Clusters of DS^MEC^ events based on CSD features. **(a)** Average LFP traces with superimposed CSD profiles for DS^MEC^ subclusters. Each panel represents a distinct cluster. **(b)** Average broadband spectrograms (1–300 Hz) are shown with log-scaled frequencies to show the low-frequency embedding for each DS^MEC^ subtype referenced to the DG granule cell layer (top) and CA1 pyramidal channel (bottom). **(c)** Heatmaps show average z-scored firing rates for DG principal cells (PC), CA1 pyramidal cells (PYR), interneurons (INT), and all cortical neurons (Ctx) recorded simultaneously in a session. For each heatmap: one cell per row, neurons were ranked (top-to-bottom) based on their respective cluster, shown on the 1st column. Only 5 of 6 DS^MEC^ clusters are shown; one cluster was discarded as falsely detected DS^LEC^ events.

To classify DS events further by their target impact, we performed unsupervised clustering using an objective Gaussian mixture model (GMM). First, DS peak CSD values across all depths (94 features) were reduced to a subset of dimensions with PCA. The number of PCs was chosen as the minimum number of components needed to capture 50% of the explained variance. Three PCs explained 53% of the total variance for DS^LEC^, while two PCs explained 60% of the total variance for DS^MEC^. Thereafter, a series of GMMs were tested, each assuming a different number of clusters present in the data (ranging from 1 to 10). The model with the lowest Bayesian information criterion (BIC) value, a metric that evaluates the model fit and complexity, determined the optimal number of clusters in a session (Supplementary Figures S11, S12; Alqahtani and Kalantan, 2020). Importantly, the BIC provides a conservative estimate of model fit by penalizing overfitting, thus favoring simpler models. The derived clusters for DS^LEC^ and DS^MEC^ are shown in Figures 8, 9.

For each cluster, we also calculated the corresponding unit firing in DG, CA1 and the overlying neocortex. The clusters were ordered (from left to right) according to the magnitude of the spike suppression preceding the LFP DS. In the DS^LEC^ group, cluster 1 and cluster 2 were similar, but cluster 2 had an additional sink at the CA1 str. Oriens/pyramidale border. In cluster 2, CA1 pyramidal cells and interneurons were more strongly recruited ~80–100 ms after the DS, compared to a weaker activation following the DS in cluster 1. Cluster 3 was preceded and followed by the largest power of gamma (50–150 Hz) oscillations, modulated by a ~ 6 Hz quasi-rhythmic firing of CA1 neurons. Cluster 4 was characterized by a delayed sink (~80–100 ms peak) in CA1 str. Radiatum. Based on the CA1 currents, cluster 4 may correspond to SPW-Rs, triggered by the CA2 and CA3 inputs to basal and apical dendrites of CA1 pyramidal cells (Lavenex and Amaral, 2000). The magnitude of neuronal suppression in the neocortex and the corresponding pre-DS suppression in the dentate and CA1 regions, preceding the peak of DSs, decreased in the cluster 1 to cluster 4 direction (Figure 9; see also Siapas and Wilson, 1998; Ji and Wilson, 2007; Swanson et al., 2025).

Six clusters were observed in the DS^MEC^ group. Two clusters corresponded to falsely detected DS^LEC^ events (not shown; dominant sink in the outer DG molecular layer) and SPW-Rs^Rad^ (Figure 9a). Cluster 1 and cluster 2 were similar in many aspects and differed only in the CSD magnitude of CA1 pyramidal currents. Both clusters exhibited activation of CA1 pyramidal cells with a ~ 80–100 ms delay relative to the DS^MEC^ peak, corresponding to an increase in ripple power in the CA1 pyramidal layer. Cluster 3 showed less pre-DS suppression of firing and was associated with lower slow power in the 1–10 Hz band. Cluster 4 had the strongest sink in the mid-molecular layer and was associated with a SPW-R^Rad^ and corresponding robust synchronous discharge of CA1 neurons. For comparison, we also show SPW-R^Rad^ detected without DSs (last panel in Figure 9). While SPW-R^Rad^ had a sink in the dentate molecular layer, as discussed above (Figure 6), this sink was deep in the supragranular layer, rather than in the mid-molecular layer (Supplementary Figure S10). Overall, the input–output classification of DSs yielded different subtypes with different features and, thus, potentially different functions.

Since DSs are driven by external inputs from the neocortex-entorhinal cortex, they depend on their dynamics. In contrast, SPW-Rs arise from internally generated mechanisms in the hippocampus when subcortical neuromodulators decrease (Zhang et al., 2021). Similar to SPW-Rs, DSs could be related to the timing of neocortical UP and DOWN states. During NREM sleep, the neocortex alternates between active (UP) and silent (DOWN) states (Steriade et al., 1993a; Steriade et al., 1993b). The UP state can spread over the entire neocortex and often invades the entorhinal cortex (Kajikawa et al., 2022; Karalis and Sirota, 2022; Levenstein et al., 2019; Nir et al., 2011; Sirota and Buzsáki, 2005). Intracellular recordings in all layers of the entorhinal cortex and dentate granule cells revealed bimodal depolarized and hyperpolarized membrane potentials, in line with the driving neocortical UP and DOWN states (Isomura et al., 2006). CA3-CA1 pyramidal cells do not display bimodal membrane potentials. Yet, their population excitability follows the neocortical UP and DOWN state (Isomura et al., 2006; Siapas and Wilson, 1998; Swanson et al., 2025). Importantly, at DOWN-UP transitions, neurons can transiently and powerfully synchronize both in the neocortex and entorhinal cortex, a pattern called K-complex (Loomis et al., 1938; Colrain, 2005). Such synchronous excitation from the entorhinal cortex can drive granule cells, resulting in a DS2. Thus, at least some DSs may be linked to neocortical-entorhinal K-complexes during NREM sleep (Sullivan et al., 2011). This can explain why some DSs (cluster 1 in Figures 7, 8) are preceded by a prominent reduction of spiking activity in the neocortex and dentate gyrus (i.e., DOWN state).

In summary, DSs differ both in their inputs (MEC and LEC) and their impact on their targets, as illustrated by the different timed firing patterns in the CA1 region. These findings can explain several inconsistencies in the descriptions of DSs reported in different papers. While the neocortical K-complex can emerge spontaneously, it was originally described as a “disturbance pattern” because it can be induced by auditory or tactile stimulation or adjustments in body position (Loomis et al., 1938). This inducibility is analogous to the evoked DS2 described by Farrell et al. (2024), and the similar depth profile of stimulus-induced startle responses and population pattern in the dentate gyrus (Brankack and Buzsáki, 1986). Furthermore, UP-DOWN state changes follow a ~ 1 min periodicity, known as NREM packets (Watson et al., 2016) or ultraslow oscillations (Penttonen et al., 1999) of sleep micro-arousals, reflected by muscular activity and K-complexes (Lüthi and Nedergaard, 2025). This relationship between K-complexes and muscular patterns is also reminiscent of the reported temporal coupling between DS2 and facial and eye movements (Farrell et al., 2024). A caveat is that most recordings in the Farrell et al. (2024), and McHugh et al. (2024) studies were performed in head-fixed mice, and their brain states were not characterized. It is thus possible that the described DS2s in both studies corresponded to an unknown combination of K-complex-triggered DSs and other events.

A fraction of DSs coincided with SPW-Rs. The coincidence occurs within a short time (<10 ms) window, making it less likely that DS induces SPW-R through activation of CA3 and CA1 circuits. One possible explanation for the coincidence is that EC2 and EC3 neurons synchronize, and EC2 population activity gives rise to a DS in the molecular layer, while EC3 induces a CA1 ripple. Under this scenario, two sinks should be observed: one in the dentate molecular layer, and the other in the CA1 str. Lacunosum-moleculare (Castelli et al., 2025). However, we have not seen this combination. Instead, SPW-R events coincided with sinks in CA1 str. Radiatum and the inner molecular layer. A reasonable interpretation then is that when a SPW-burst occurs in CA3, the CA3 pyramidal cells induce a SPW in CA1 str. Radiatum and the collaterals of CA3 neurons activate mossy cells, and, in turn, a sink in the inner molecular layer (Scharfman, 1994, 2007; Sullivan et al., 2011). This scenario is supported by our observation that the dentate sink associated with SPW-Rs was deeper, corresponding to the supragranular layer (Figure 9), rather than the mid-molecular layer, the main input from MEC. Reliably identifying sinks in the inner, mid, and outer thirds of the molecular layer requires high-density (<30 μm) spacing of linear probe recording sites. Simultaneous recording of MEC and LEC layer 2 and layer 3 neurons, together with linear probe recordings in the dentate-CA1 axis, will be required to pin down the exact origin of the outer- and mid-molecular layer sinks.

A subset of DSs (clusters 1 and 2 of DS^MEC^ and clusters 2 and 3 of DS^LEC^) were followed by CA1 ripples with an 80–100 ms delay, implying that occasionally DSs can induce SPW-Rs. This combination may correspond to SPW-R_DU_ events that occur ~120 ms after neocortical DOWN-UP transition (Swanson et al., 2025). The variable relationship between SPW-Rs and DSs may explain why the timing relationship between DSs and SPW-Rs has been controversial in different publications. Further work, comparing the different DS subgroups in learning situations, is needed to uncover how DSs can bias SPW-Rs and how their spike content relates to the spike content of SPW-Rs.

## Conclusion

We have described the main characteristic LFP patterns in the hippocampus, including SPW-Rs, DSs, theta, and gamma oscillations. Several of our observations confirm and extend previous analyses (Berényi et al., 2014; Bragin et al., 1995a, 1995b; Buzsáki, 2002; Buzsáki et al., 1985; Fernández-Ruiz et al., 2021; Lubenov and Siapas, 2009; Farrell et al., 2024; McHugh et al., 2024; Montgomery et al., 2008; Oliva et al., 2016a; Sullivan et al., 2011; Winson, 1974). Our novel observations relate mainly to the distinction of subclasses of SPW-Rs and DSs and their neuronal spiking correlations. In addition to the classical SPW-Rs, initiated in the CA2-3 recurrent collateral system and characterized by a large negative sharp wave (sink) in the mid-CA1 stratum radiatum (SPW-R^Rad^), a small subset of ripples, associated with a sink in the CA1 str. Lacunosum-moleculare were also observed (SPW-R^LM^). The two types of ripple events differed in frequency, magnitude, and neuronal correlates. CA3 pyramidal neurons were strongly active during SPW-R^Rad^ but not during (SPW-R^LM^; Castelli et al., 2025). DSs could also be grouped further, based on their inducing inputs, as DS^MEC^ and DS^LEC^, by their impact on their physiological targets and by the brain states into which they were embedded. Overall, our experiments demonstrate the utility and need for high-density recording of both LFP and spiking activity for the appropriate classification of seemingly similar events. These distinctions relate not only to their neurogenesis but also to their behavioral-cognitive contributions.

## Data Availability

The datasets presented in this study can be found in online repositories. The names of the repository/repositories and accession number(s) can be found in the article/Supplementary material.

## Footnotes

^1^ https://github.com/tortlab/dentate-spikes

^2^ https://phy-contrib.readthedocs.io/

^3^ https://cellexplorer.org/pipeline/cell-type-classification/
