# Supplementary material for: Electroanatomy of hippocampal activity patterns: theta, gamma waves, sharp wave-ripples, and dentate spikes
Source: Front Behav Neurosci. 2025 Oct 23;19:1685846. doi: 10.3389/fnbeh.2025.1685846 (PMC12589087; doi:10.3389/fnbeh.2025.1685846)
Supplement: Supplementary file 1 [file Data_Sheet_1.docx]

**Supplementary Figures**

**
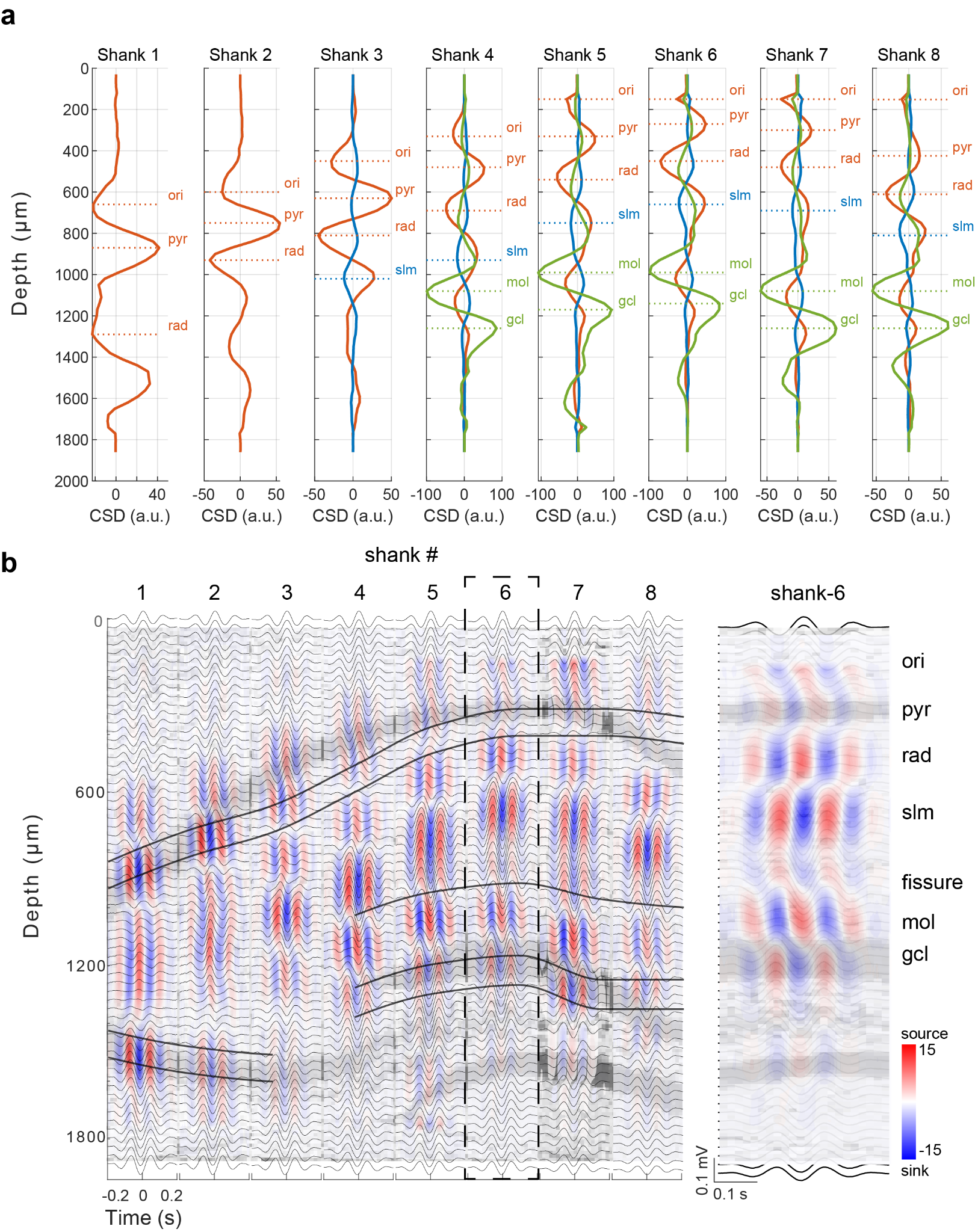
**

**Figure S1. Hippocampal layer identification across 8 shanks. (a)** Representative channels in each hippocampal layer identified by CSD features in the average SPW-R (120-200 Hz, red), theta (5-12 Hz, blue), and dentate spike (1-200 Hz, green) linear CSD profiles across shanks. **(b)** Average theta-triggered CSD map aligned to the detected pyramidal layer (pyr) for each shank. Color scale represents the CSD derived from the second spatial derivative of the LFP across depths. The electrophysiologically-defined anatomical outlines are superimposed on a dorsal hippocampal section stained with DAPI (dark) to demonstrate the approximation of the hippocampal cell body layers by LFP features. Ori: oriens; rad: radiatum; slm: lacunosum-moleculare; mol: DG molecular layer; gcl: DG granule cell layer.

***
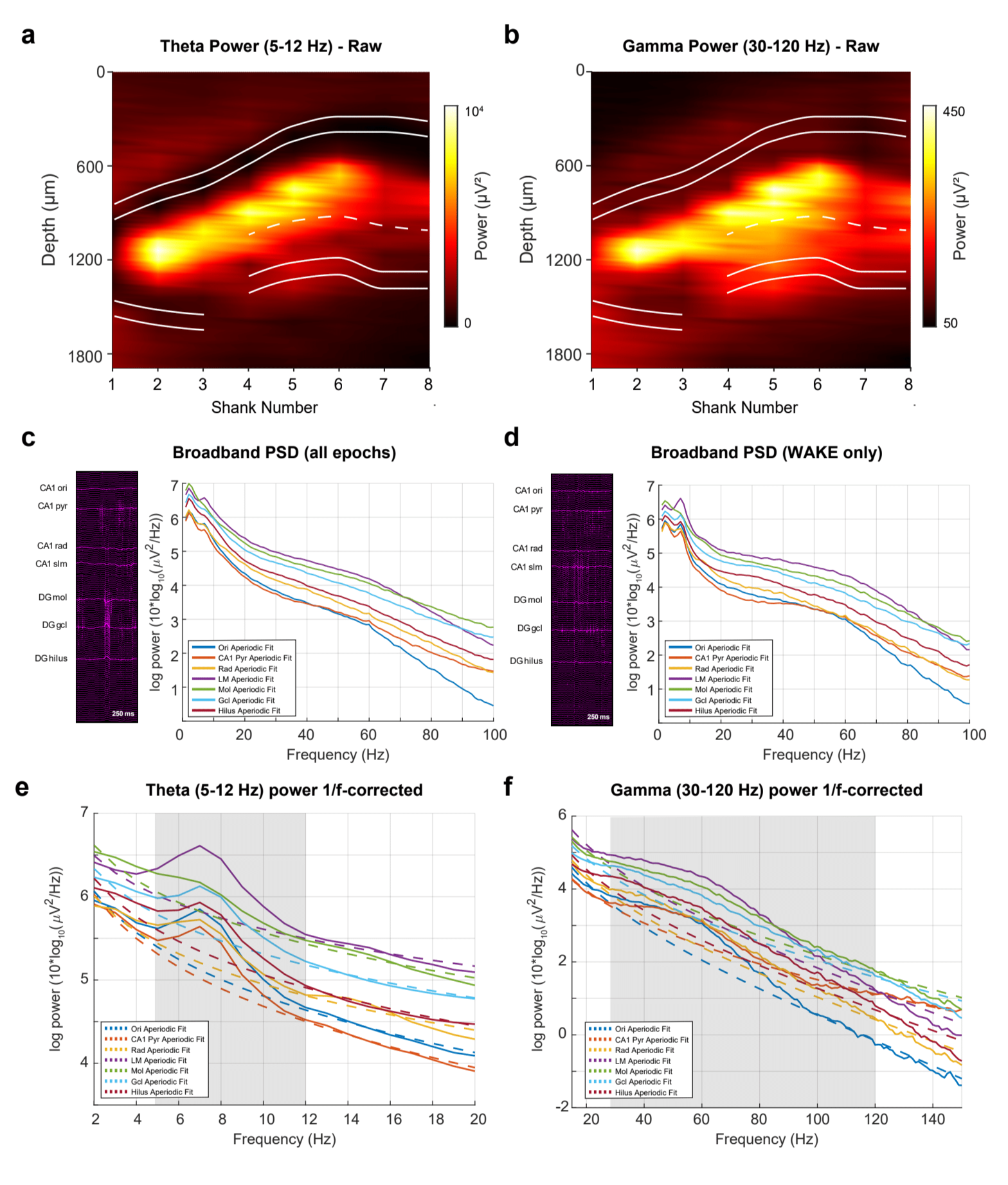
***

**Figure S2. Fitting 1/f to the theta and gamma power spectra.** Raw theta (a) and gamma (b) power maps were computed during wake epochs using Welch’s method. Although the data used in Fig. 4 are identical, the power maps presented here did not undergo 1/f correction. (c, d) Unfiltered snippets (i.e., pior to LFP downsampling; 250 ms) from shank 6 of the recording used for power analysis, ordered by increasing depth from the brain surface (left). Thickened purple lines highlight the channels used for power spectral density (PSD) analysis. PSD plots are shown (right) for a representative SiNAPS session (60.2 min duration); PSDs were computed separately for all epochs (c) and awake periods (d; 21.1 min). (e, f) The FOOOF toolbox (Donoghue et al., 2020) was used to fit a line to the aperiodic component of each PSD plot presented in log scale. Theta (e) and gamma (f) frequency ranges were fitted separately. Each color-coded (see legend) PSD plot from panels c-f was computed with the LFP from contacts on shank 6 (spanning the distal CA1-DG axis), corresponding to distinct hippocampal layers as defined by the electrophysiological landmarks described in Fig. 1c. Solid lines represent the raw power spectra while dotted lines of the same color correspond to the aperiodic fit obtained by 1/f-correction. Note the large shifts in the direct current (DC), or aperiodic, component of the power spectrum across hippocampal layers. Thus, 1/f-correction of PSD plots is critical for directly comparing power in frequency bands of interest across hippocampal depths, minimizing the impact of systematic DC shifts on power calculations.

**
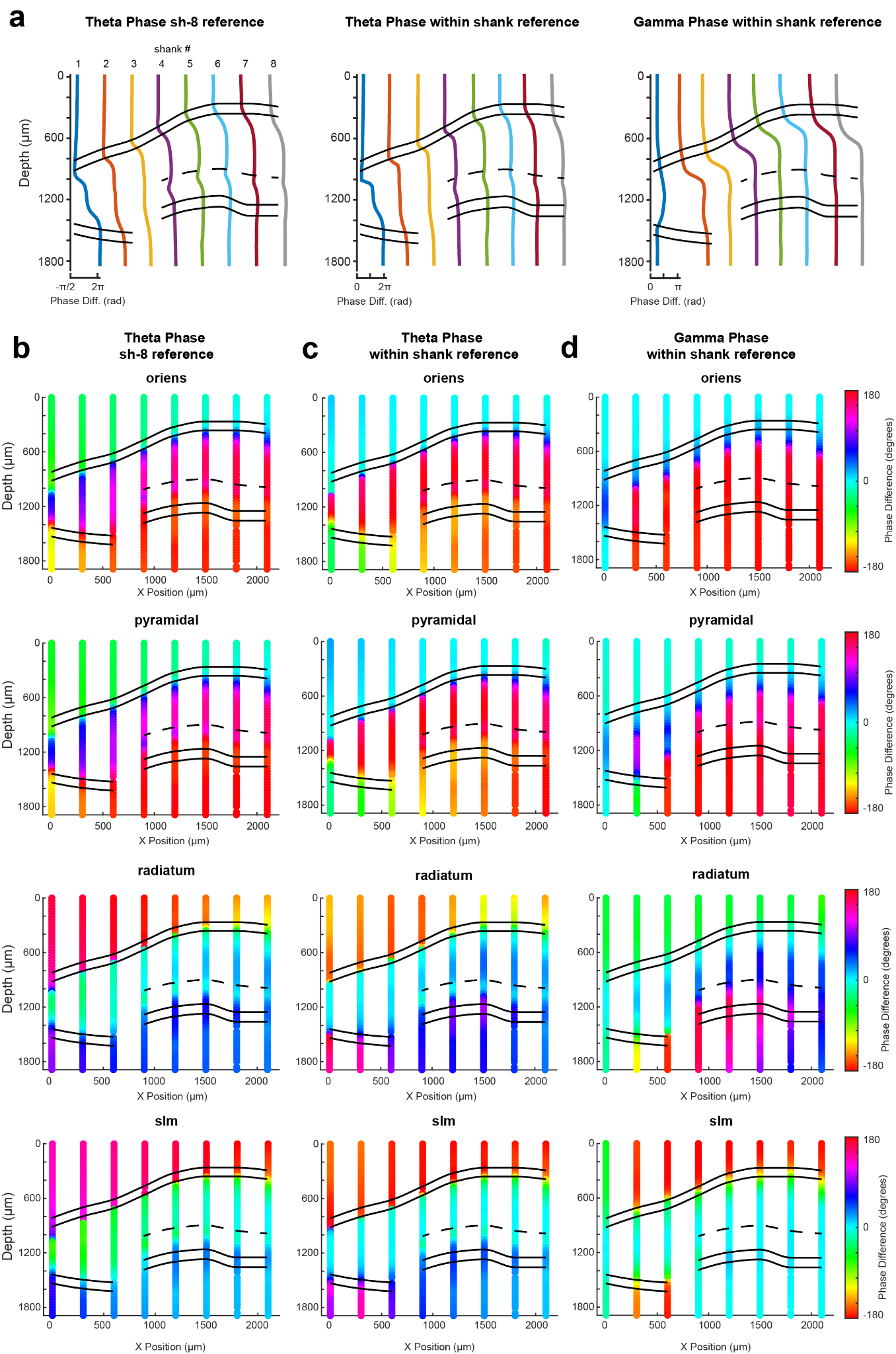
**

**Figure S3. Theta and gamma phase shifts across the hippocampal transverse axis.** **(a)** Phase differences across the dorsal hippocampus referenced to theta (5-12 Hz) peaks in the distal CA1 (shank 8) str. oriens channel (left) and str. oriens channel on each shank (middle); similarly, gamma (30-120 Hz) phase changes across depths are shown referenced to gamma peaks in the str. oriens on each shank (right). Average instantaneous phase differences were extracted for each channel by applying the Hilbert transform to the theta or gamma bandpass-filtered LFP during wake epochs. Note that each panel has a distinct scale of phase differences. **(b-d)** Theta and gamma phase maps referenced to distinct CA1 sublayers. Phase differences are represented by a circular color scale. Cyan indicates no phase shift, while red reflects a phase reversal of 180 degrees relative to a single reference (left column) or within-shank reference sites (middle and right columns).

**
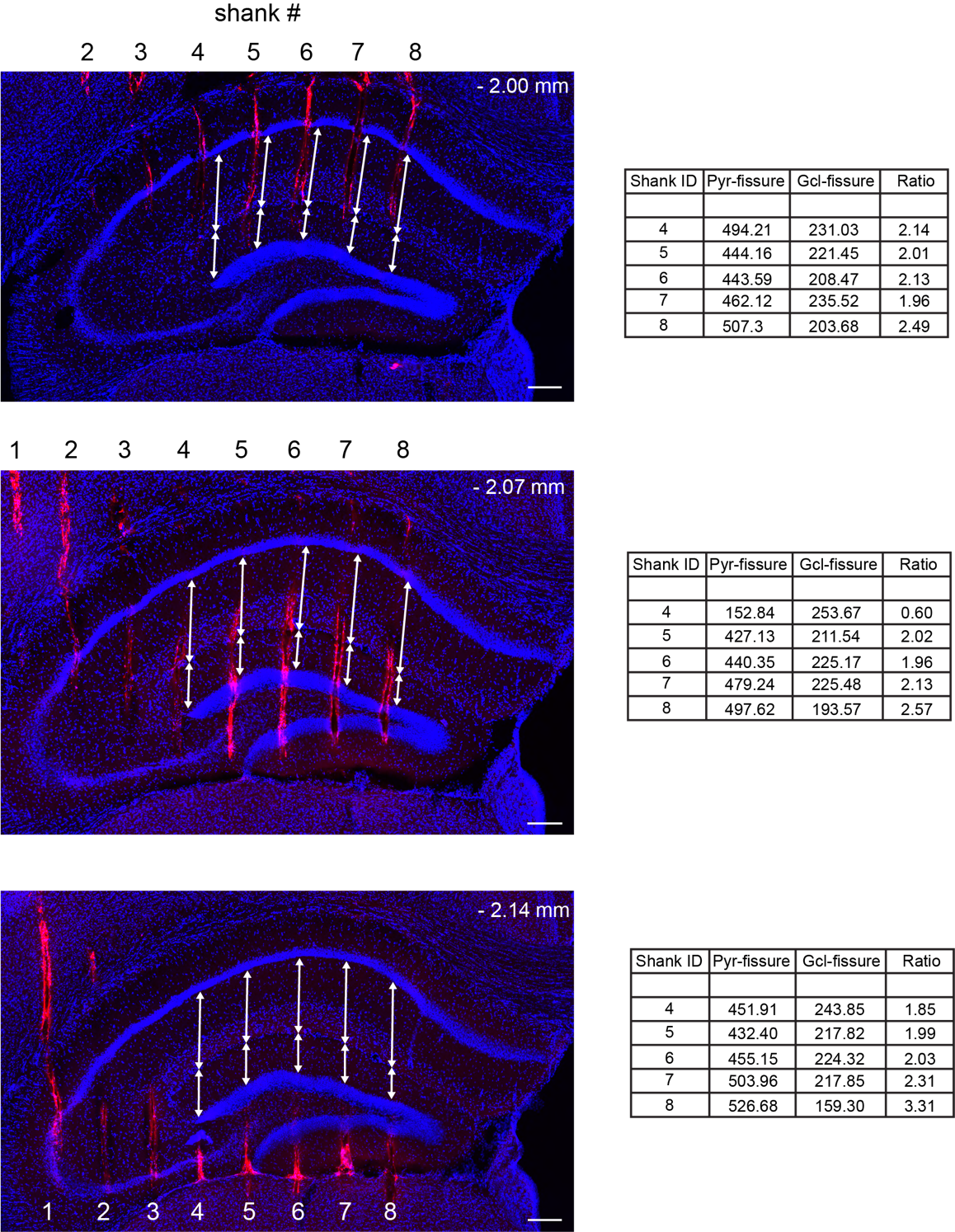
**

**Figure S4. Histological evaluation of hippocampal fissure location.** Serial coronal sections of the mouse hippocampus, stained with DAPI (blue), highlighting the cell bodies, and DiI (red), reflecting the tracks of each shank of the 8-shank SiNAPS probe. For each section, the distance between the CA1 pyramidal cell layer to the hippocampal fissure (Pyr-fissure) and the distance between the fissure and granule cell layer (Gcl-fissure) are quantified. The ratio between these distances is shown for each shank spanning the fissure (shanks 4-8). The average ratios across serial sections for each shank were 1.53 (shank 4), 2.0 (shank 5), 2.04 (shank 6), 2.13 (shank 7), and 2.79 (shank 8).


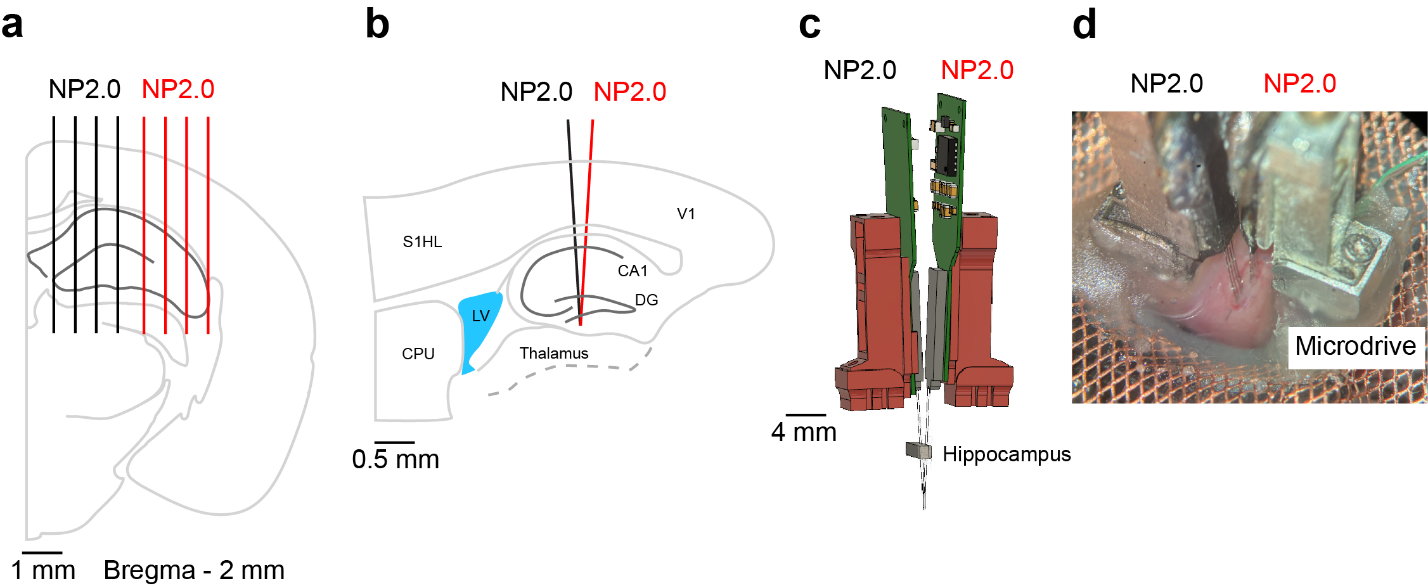


**Figure S5. Dual probe Neuropixels recording from the transverse axis of the hippocampus. (a)** Schematic of implantation of the Neuropixels probes (coronal view). Mouse was implanted two probes (medial probe is black, lateral probe is red). **(b)** Probes were implanted at 2 degrees angle to cross each other in dentate gyrus (sagittal view). **(c)** CAD rendering of probes attached to microdrives targeting hippocampus. **(d)** Intraoperative photograph shows the two probes inserted into the brain. Microdrives are attached to the skull. Craniotomy is covered with duragel.


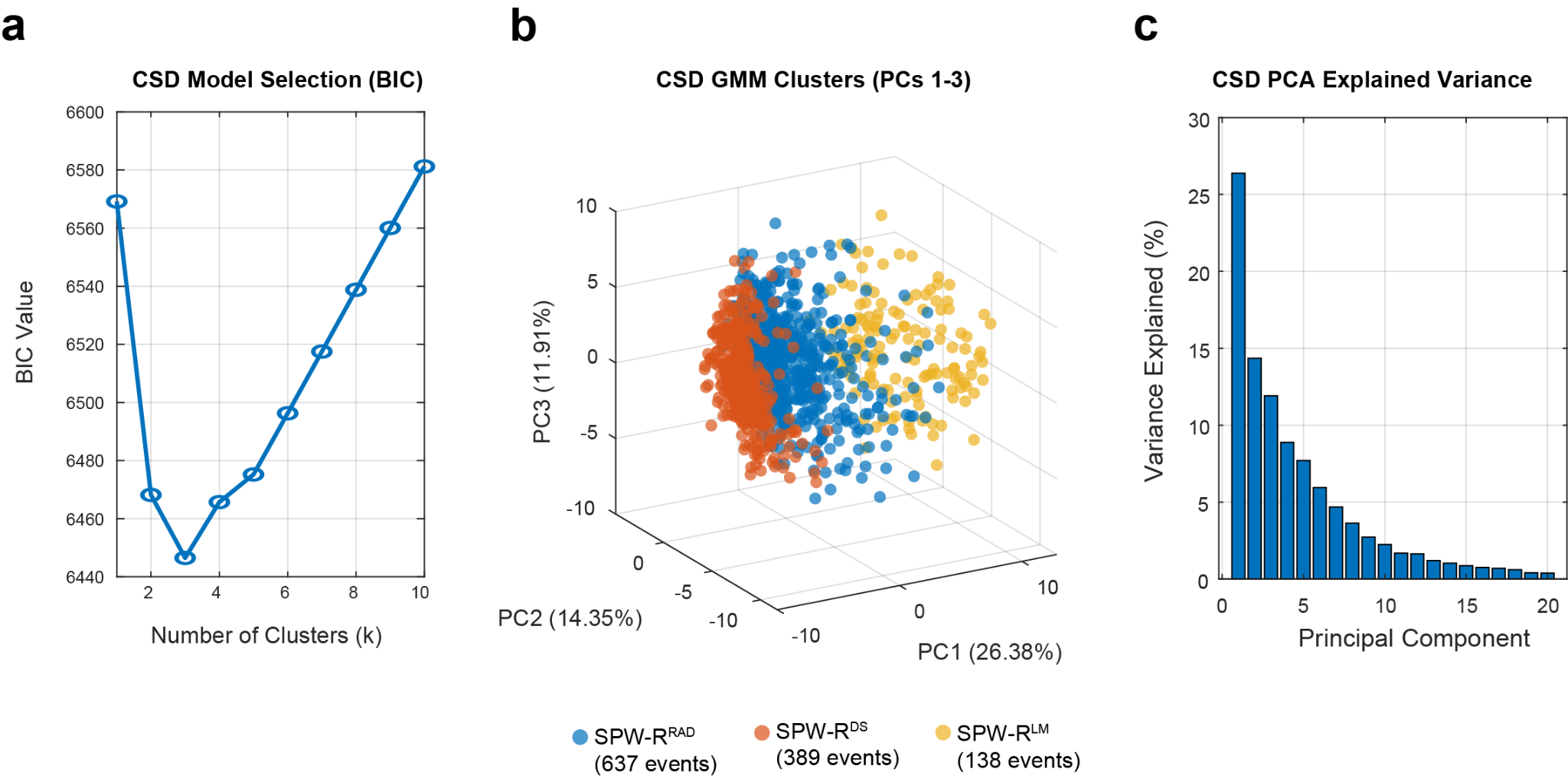


**Figure S6.** **Determining the number of CSD-based SPW-R clusters. (a)** Bayesian information criterion (BIC) values are shown for a set of GMMs assuming a different number of clusters (k) present in the data. The trough in the BIC curve reflects the optimal number of SPW-R clusters based on their individual CSD profiles, indicating that the GMM with 3 clusters fits the data best in this representative session. **(b)** Each data point represents a feature vector composed of CSD values obtained at the peak timestamp for each event. The data points are colored based on the cluster ID determined by the BIC-optimized GMM. Here PCs 1-3 are shown, although only the first PC was used for SPW-R event clustering since this consistently yielded the separation of ripple events with a dominant sink in the str. radiatum and another group in the str. lacunosum-moleculare. **(c)** Scree plot of the top 20 PCs, demonstrating how much of the total variance in the data is explained by each PC.


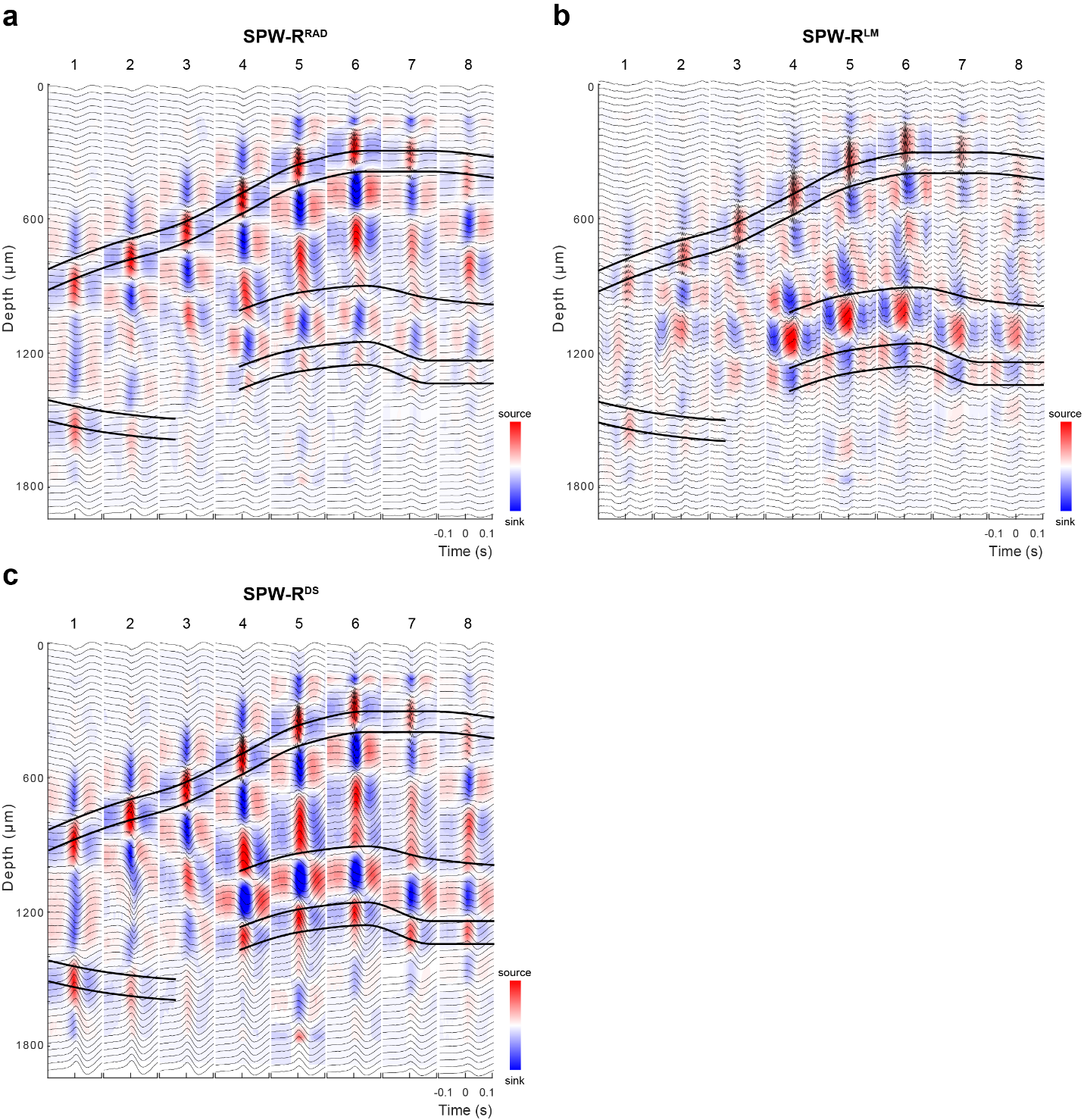


**Figure S7. CSD of different SPW-R types across the transversal axis of the hippocampus.** Average CSD of the different ripple types overlaid with average LFP (grey lines) recorded across 1024 channels. Blue and red represent current sink and source, respectively. 0 µm represents the most dorsal channel on the probe. **a)** SPW-R^Rad^ events. **b)** SPW-R^LM^ events. **c)** SPW-R^Rad^ associated with a DS (SPW-R^DS^ events).

**
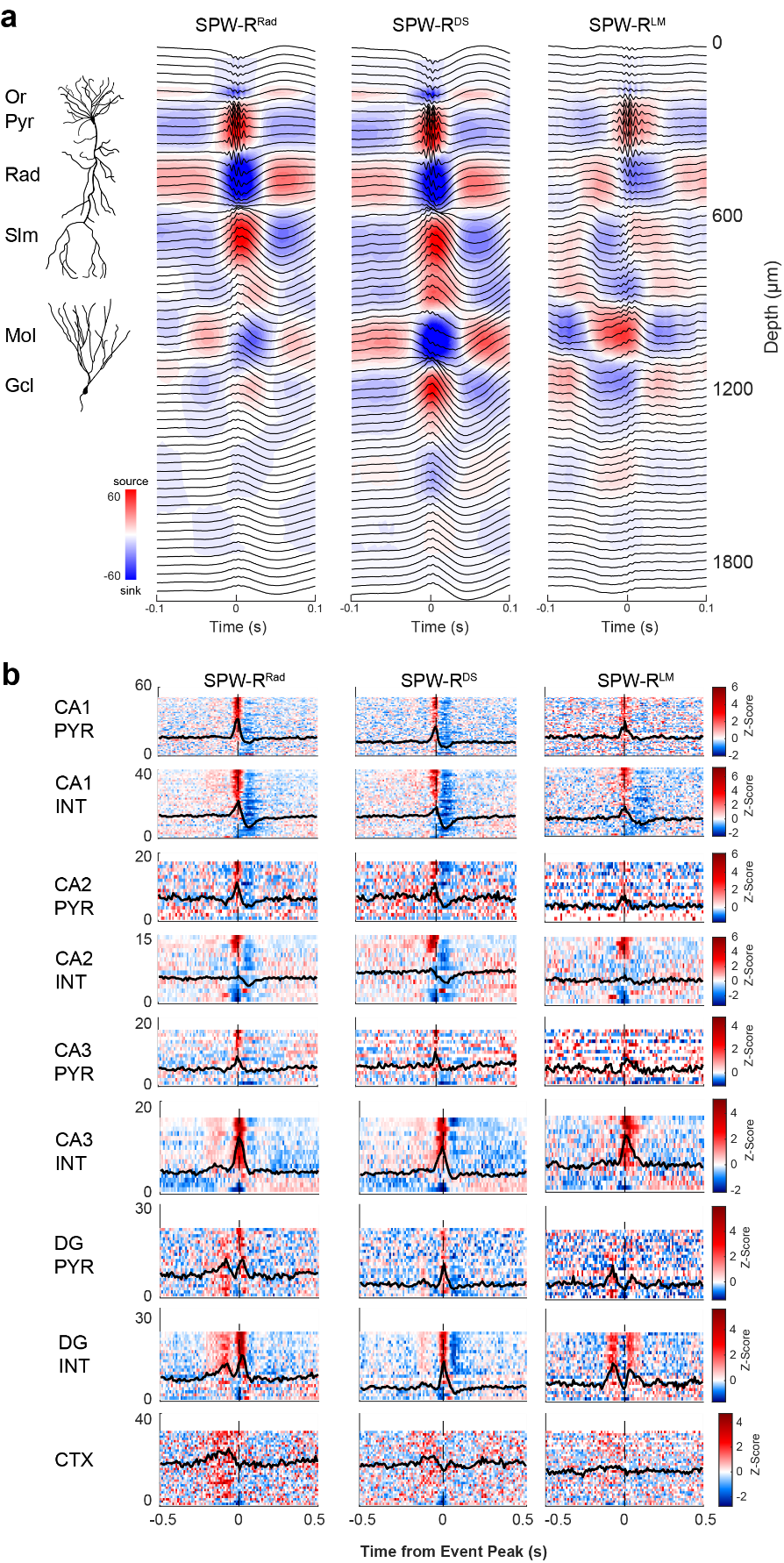
**

**Figure S8. CSD profiles and cell-type–specific firing during distinct SPW-R subclasses. a)** Average CSD maps for three identified SPW-R types: SPW-R^RAD^, SPW-R^DS^, and SPW-R^LM^. Depth is aligned to hippocampal layers. Red indicates current sources and blue indicates sinks, overlaid on LFP traces across probe recording sites. **b)** Z-scored peri-event time histograms of firing rates for pyramidal cells (PYR) and interneurons (INT) in CA1, CA2, CA3, dentate gyrus (DG), and neocortex (CTX) aligned to the peak of each SPW-R type. Black lines indicate average firing probability; red/blue colors indicate deviations from baseline (red: increased firing, blue: decreased firing).


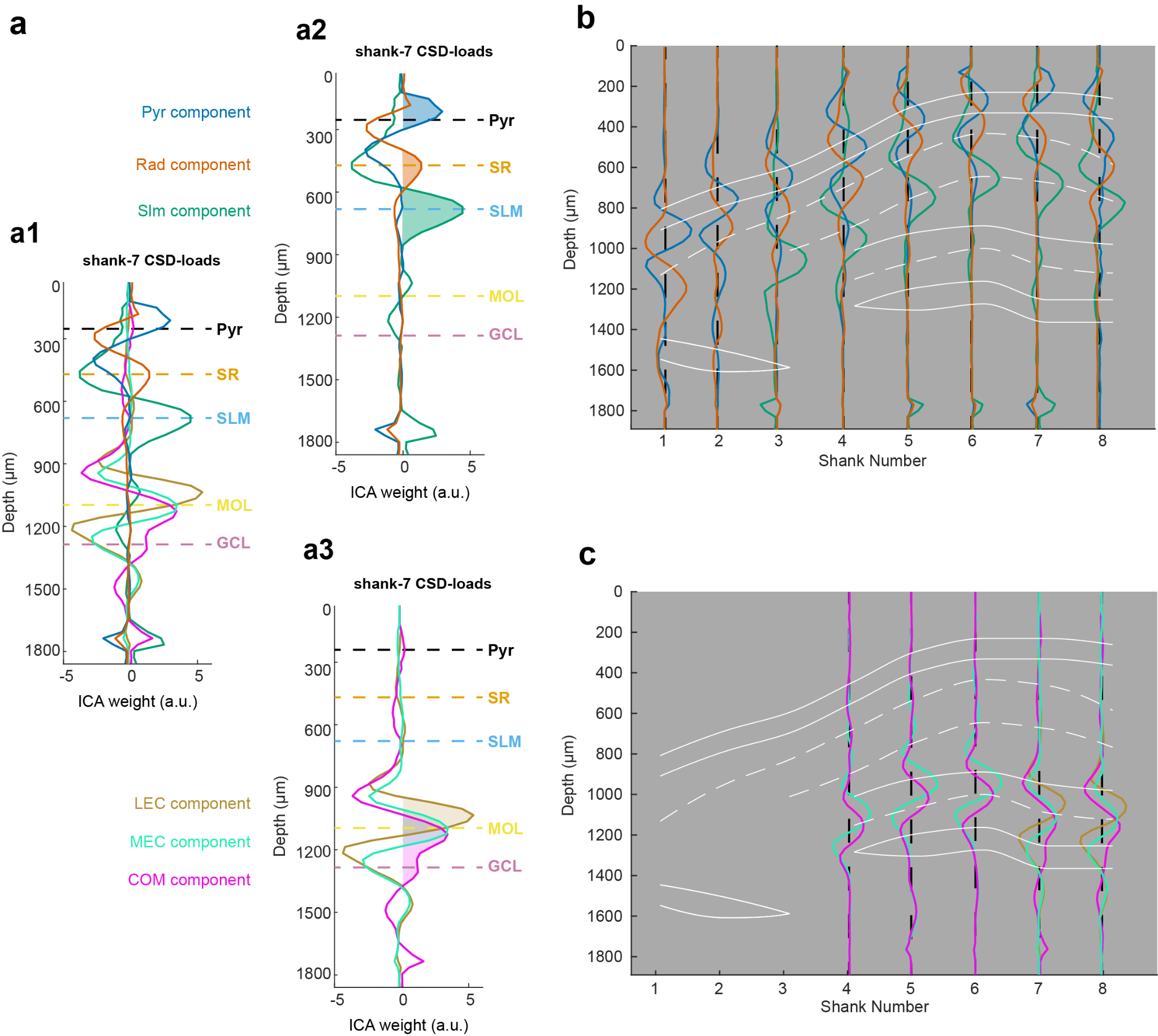


**Figure S9. ICA decomposition of LFPs along the dorsoventral hippocampal axis. a1)** ICA decomposition of LFPs into 6 main components (ICs). The CSD load of the CA1pyr ICs (blue trace) peaked at the str. pyramidale (Pyr, black dashed line). The rad IC (orange) peaked in the str. radiatum (SR, orange dashed line). The slm IC (dark green) identified the str. lacunosum-moleculare (SLM, light blue dashed line). The molecular ICs (brown, turquoise, purple) peaked in the molecular layer (Mol, yellow dashed line), reflecting separation of LEC, MEC and commissural (COM) inputs received by the granule cells. **a2)** Three components separating the sublayers of CA1 on an example shank (shank-7). **a3)** Three components separating the sublayers of DG from CA1 on an example shank (shank-7). **b)** CA1 components across all shanks overlaid on the hippocampal anatomy (white dotted, dashed and solid lines). **c)** DG components across all shanks overlaid on the hippocampal anatomy (white dotted, dashed and solid lines).


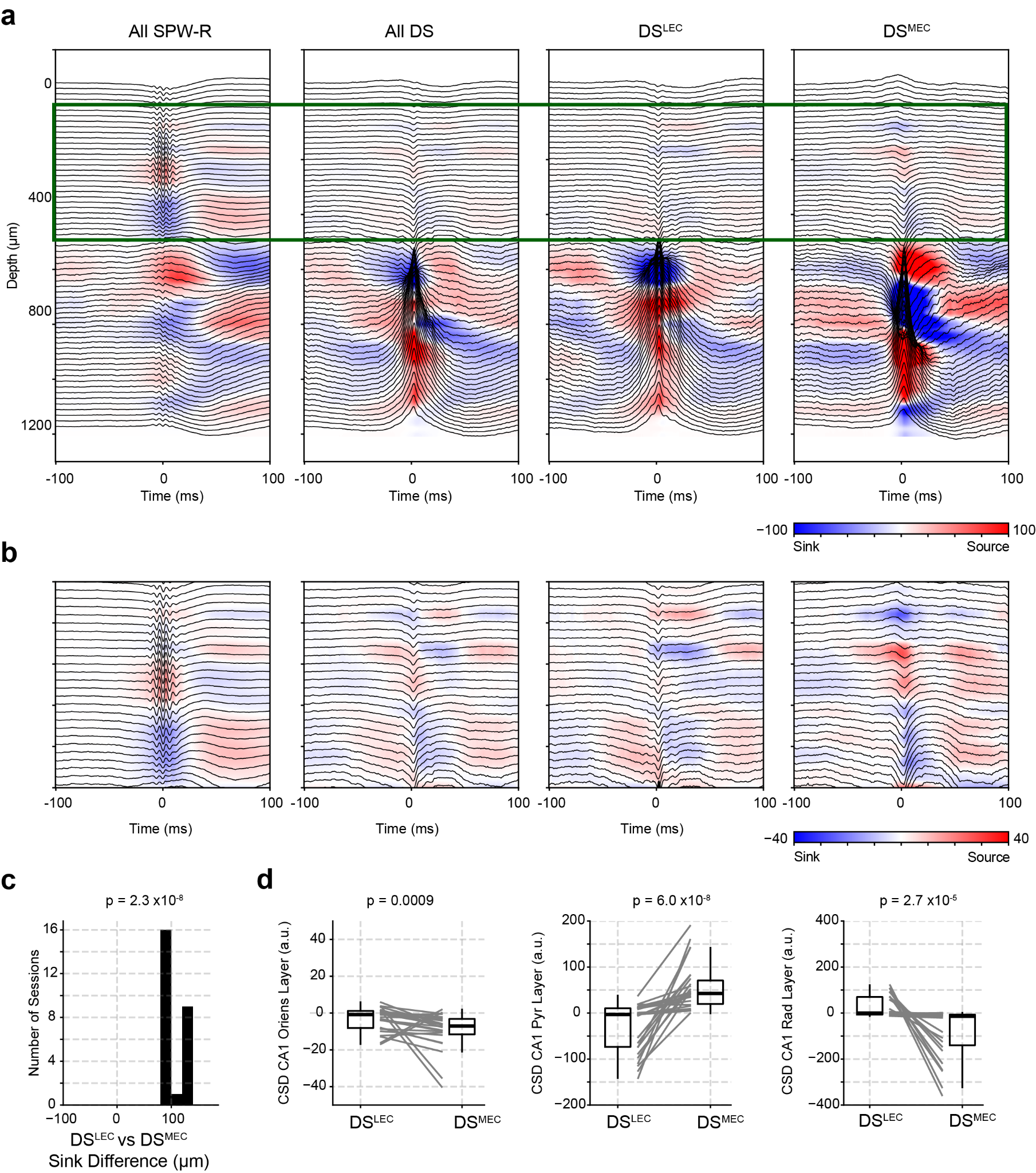


**Figure S10. Distinct dentate spike types evoke different dentate and CA1 current source–sink profiles. (a)**Example average CSD traces aligned to event peak (time = 0 ms) across hippocampal depth for SPW-Rs and for DS^LEC^ and DS^MEC^ (DS1 and DS2, respectively). Black traces show the averaged raw LFPs; colored overlays show CSD maps (blue: sinks, red: sources). **(b)**Zoomed-in CSD plots in the CA1 region. Note different sink-source patterns during DS^MEC^ and DS^LEC^. **(c)**Histogram of within-session differences in peak sink amplitude between DS^LEC^ and DS^MEC^ events, revealing consistently deeper sink for DS^MEC^ than DS^LEC^ across sessions (p = 2.3 × 10⁻⁴, sign-rank test, n = 26 sessions in 4 mice). **(d)** Quantification of peak CSD amplitude during DS^LEC^ vs. DS^MEC^ events in CA1 str. oriens (left), str. pyramidale (middle), and str. radiatum (right) layers across sessions. p values were obtained with a sign-rank test, n = 26 sessions in 4 mice. (Data from Zutshi et al., 2022).


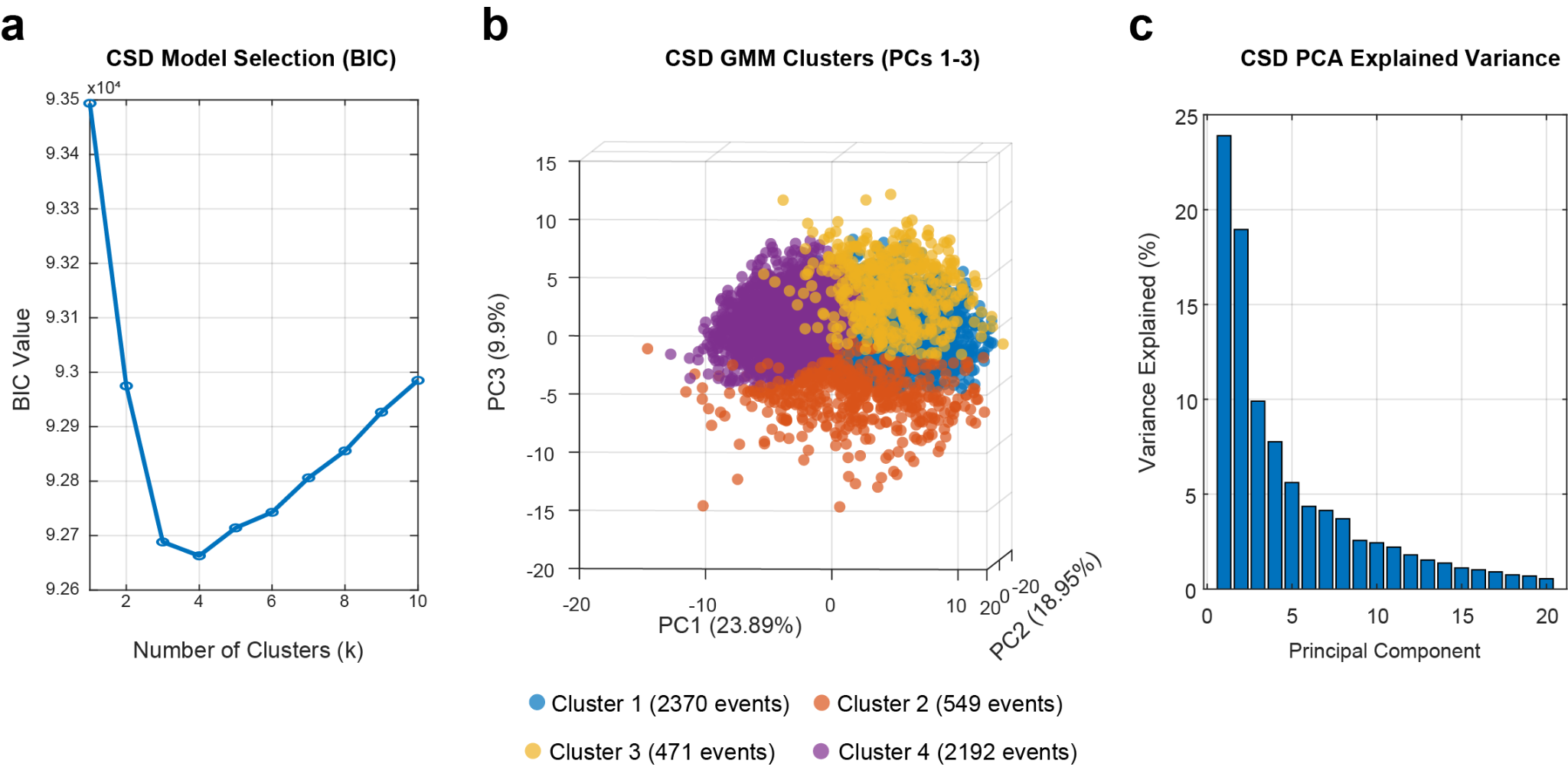


**Figure S11. Determining the optimal number of DS^LEC^ clusters. (a)** BIC values are shown for a set of GMMs assuming a different number of clusters (k) present in the data. The trough in the BIC curve reflects the optimal number of DS^LEC^ clusters based on their individual CSD profiles, indicating that the GMM with 4 clusters fits the data best in this session. The sessions chosen for DS analysis were from chronically implanted, freely moving mice with ~2h of NREM sleep, when DSs dominate. **(b)** Each data point represents a feature vector composed of CSD values obtained at the peak timestamp for each event. The data points are colored based on the cluster ID determined by the BIC-optimized GMM. The top 3 PCs were used for event clustering to capture at least 50% of the explained variance in the data **(c)** Scree plot of the top 20 PCs, demonstrating how much of the total variance in the data is explained by each PC.

**
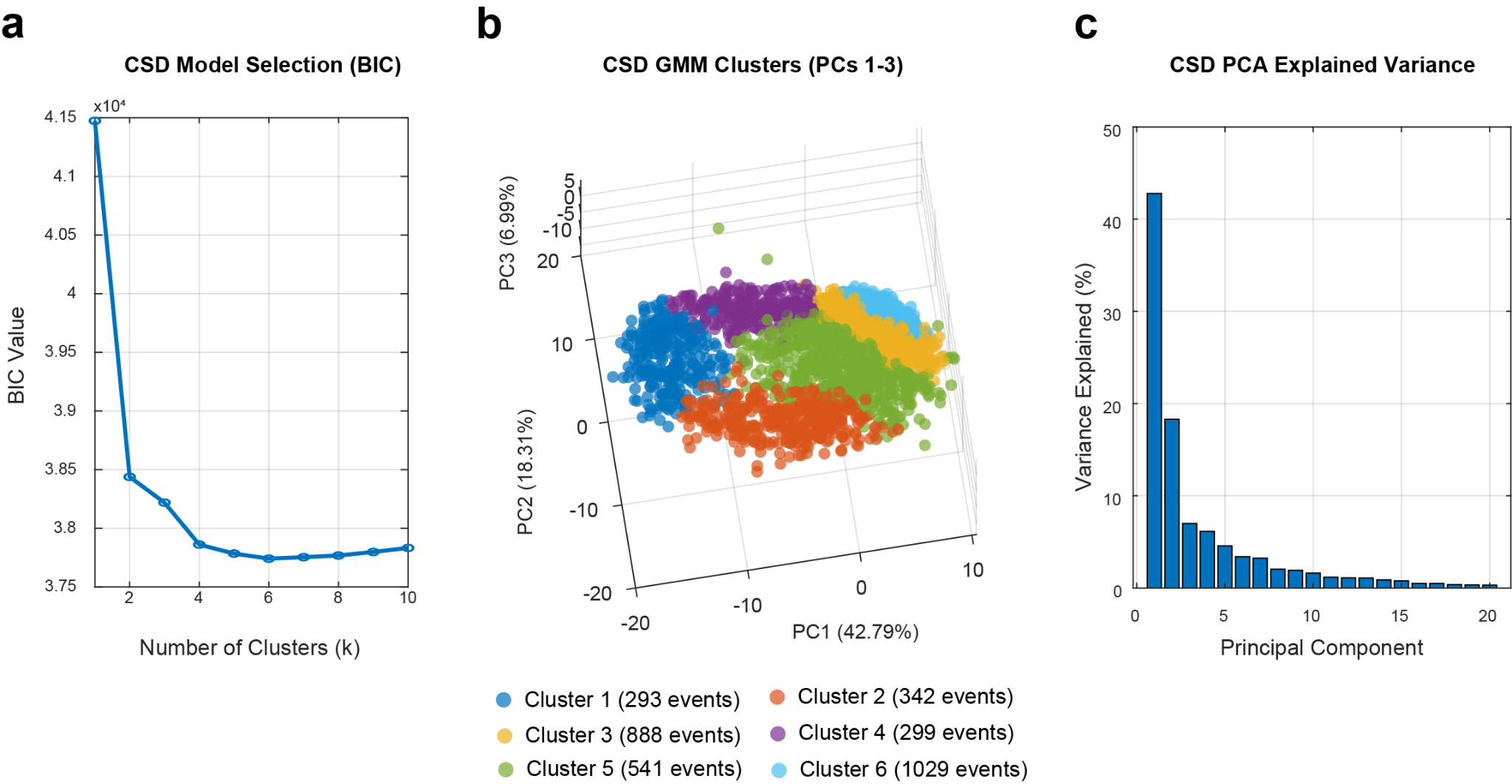
**

**Figure S12. Determining the optimal number of DS^MEC^ clusters. (a)** BIC values are shown for a set of GMMs assuming a different number of clusters (k) present in the data. The trough in the BIC curve reflects the optimal number of DS^MEC^ clusters based on their individual CSD profiles, indicating that the GMM with 6 clusters fits the data best in this session. The sessions chosen for DS analysis were from chronically implanted, freely moving mice with ~2h of NREM sleep, when DSs dominate. **(b)** Each data point represents a feature vector composed of CSD values obtained at the peak timestamp for each event. The data points are colored based on the cluster ID determined by the BIC-optimized GMM. The top 2 PCs were used for event clustering to capture at least 50% of the explained variance in the data **(c)** Scree plot of the top 20 PCs, demonstrating how much of the total variance in the data is explained by each PC.
